# A reappraisal of the uncanny valley: categorical perception or frequency-based sensitization?

**DOI:** 10.3389/fpsyg.2014.01488

**Published:** 2015-01-21

**Authors:** Tyler J. Burleigh, Jordan R. Schoenherr

**Affiliations:** ^1^Department of Psychology, University of GuelphGuelph, ON, Canada; ^2^Department of Psychology, Carleton UniversityOttawa, ON, Canada

**Keywords:** uncanny valley, categorical perception, category learning, categorization, exemplar theory, exemplar-based, frequency-based, affect

## Abstract

The uncanny valley (UCV) hypothesis describes a non-linear relationship between perceived human-likeness and affective response. The “uncanny valley” refers to an intermediate level of human-likeness that is associated with strong negative affect. Recent studies have suggested that the uncanny valley might result from the categorical perception of human-like stimuli during identification. When presented with stimuli sharing human-like traits, participants attempt to segment the continuum in “human” and “non-human” categories. Due to the ambiguity of stimuli located at a category boundary, categorization difficulty gives rise to a strong, negative affective response. Importantly, researchers who have studied the UCV in terms of categorical perception have focused on categorization responses rather than affective ratings. In the present study, we examined whether the negative affect associated with the UCV might be explained in terms of an individual's degree of exposure to stimuli. In two experiments, we tested a frequency-based model against a categorical perception model using a category-learning paradigm. We manipulated the frequency of exemplars that were presented to participants from two categories during a training phase. We then examined categorization and affective responses functions, as well as the relationship between categorization and affective responses. Supporting previous findings, categorization responses suggested that participants acquired novel category structures that reflected a category boundary. These category structures appeared to influence affective ratings of eeriness. Crucially, participants' ratings of eeriness were additionally affected by exemplar frequency. Taken together, these findings suggest that the UCV is determined by both categorical properties as well as the frequency of individual exemplars retained in memory.

## Introduction

Categorization is a critical determinant of human survival. In the absence of categories, humans would be required to learn whether each stimulus that we encountered was desirable or noxious as well as whether the conspecifics that we encountered were kin or competitors. The variability in cross-cultural folktaxonomies (Medin and Atran, 1999), color classification (Regier and Kay, 2009), and speech perception (Pisoni et al., 1982) demonstrates that while humans might have prepotent responses to ranges of stimuli, many of these distinctions can be modified or must be learned. When available within the classification system of society, these categories can be associated with strong, negative affect responses (Schoenherr and Burleigh, 2014). Thus, categories both reflect and determine one's experience of the world.

Group membership and identity form an especially relevant class of categories for humans (for a review, see Fiske and Taylor, 2013). In the social context, repeated exposure to individuals within a group can increase affiliation and conformity (for review, see Bond and Smith, 1996) among group members while also leading to negative affective responses toward out-group members (for review, see Cialdini and Goldstein, 2004). This suggests the possibility that mixing features that have strong associations with members of contrasting categories will either lead to a reduction in positive affect or an increase in negative affect (Burleigh et al., 2013). In contrast to categorical perception, sub-categorical properties such as exposure to individual exemplars has long been considered an important determinant of affective responses (e.g., Fechner, 1876; Maslow, 1937; Zajonc, 1968). The present study considers how the comparatively low frequency of exposure to stimuli selected from a region of a continuum can lead to negative affective responses. We examine this in the context of negative affective responses to stimuli containing features from contrasting categories.

In the context of human factors, Mori's (1970) Uncanny Valley Hypothesis (UVH) suggests that human-like objects in our environment might come to be associated with negative affect if they possess a certain degree of human-likeness. Recently, a number of authors have suggested potential explanations of the UVH that are either explicitly or implicitly based on categorical perception (Cheetham et al., 2011, 2013; Moore, 2012; Burleigh et al., 2013; Yamada et al., 2013; Ferrey et al., submitted). While these studies have made important theoretical contributions, the implications of different learned category representations on the UCV phenomenon have not been directly tested. In the present study, we sought to address this gap by using a category-learning paradigm in which groups of participants received different sets of training stimuli consisting of computer-generated creatures. We examine participants responses to creatures following training, specify the conditions in which affective minima associated with the UCV would be observed, the properties of category learning that would determine the location of affective minima, and what underlying representation of category structures would best fit the response patterns that we observed.

### The uncanny valley

**Figure 1:**
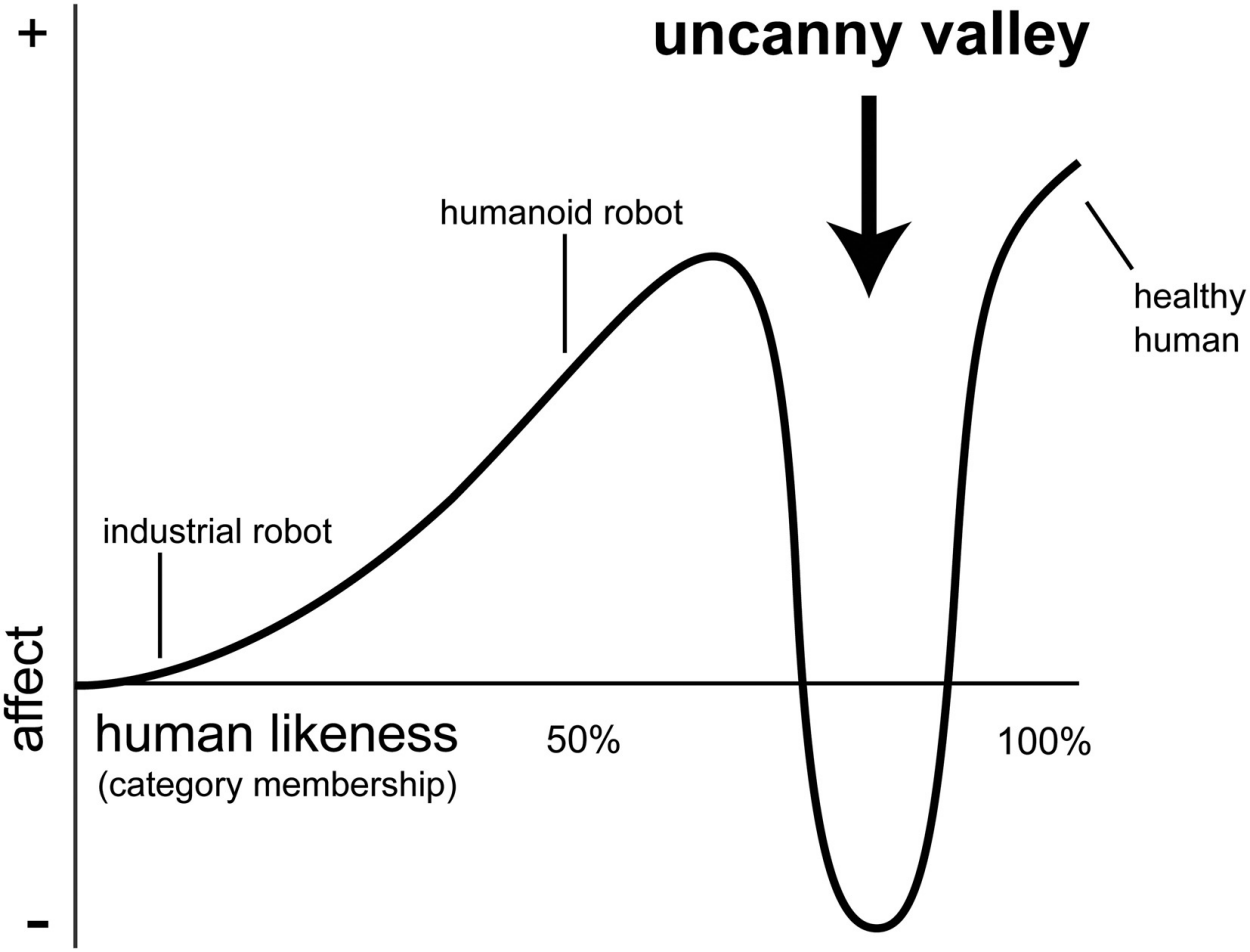
**The N-Shaped Uncanny Valley function, as proposed by Mori (1970)** (illustration adapted from MacDorman, 2005).

While the essential phenomenon of the uncanny valley has a number of cultural antecedents (Schoenherr and Burleigh, 2014), the Uncanny Valley Hypothesis (UVH) remains underspecified. Mori (1970) initially proposed that human-like stimuli can elicit positive or negative feelings depending on their degree of similarity to humans. In contrast to the linear relationship between familiarity and positive affect for human and human-like faces (see Experiment 1, Burleigh et al., 2013), the UVH predicts a non-linear relationship. Mori's account assumes that as stimuli become defined by more human-like features, they will elicit greater positive affect. But importantly, his account also assumes that there is a critical region of intermediate human-likeness where a sharp decrease in positive feelings are observed. As illustrated in Figure 1, the proposed relationship resembles a cubic function, and the global minimum is referred to as the “valley.”

It is important to distinguish between the classic and generalized forms of the UVH. The classic account of the UVH provided by Mori (1970) is defined as a non-linear function. In his account, the x-axis of this function is defined as human-likeness, and it is anchored by a non-human or minimally human-like entity at one end (e.g., a robot) and a real human at the other end. One reason to question this account is that it was informed by anecdotal evidence in the context of the human-like design of machines and other artifacts. The basic premise that negative affect could be a consequence of mismatch between features associated with contrasting classes would explain many cross-cultural phenomena (Schoenherr and Burleigh, 2014). In contrast to this, the UVH could be taken as assuming that the non-linear response function observed with human-likeness is a special case of more general cognitive and affective processes associated with stimulus frequency and categorical perception. Thus, it follows that similar non-linear phenomena should be observed in response to perceptual continua that represent non-human anchors with similar properties.

Evidence that has been interpreted as supporting the classic UVH has been obtained from studies using a variety of stimuli selected from a number of ontological categories. A majority of these studies have observed affective functions that are consistent with the UVH when using stimuli representing computer-generated morph sequences of human and non-human entities, including non-human animals, robots, and anthropomorphic dolls (MacDorman and Ishiguro, 2006; Seyama and Nagayama, 2007; Burleigh et al., 2013; Ferrey et al., submitted). Many studies have also observed the affective function in response to images of existing artifacts that vary in human-likeness, such as androids, videogame characters, and prosthetic hands (Bartneck et al., 2007; Schneider et al., 2007; Poliakoff et al., 2013); however, it is worth noting that several studies have not found support for the classic account of the UVH (MacDorman et al., 2009; Cheetham et al., 2014). Across those studies which have found support, a general observation is that affective response is positively correlated with human-likeness, except at an intermediate level of human-likeness where there is a maximum of negative affect.

Few studies have examined the possibility that perceptual continua representing non-human entities could produce UCV phenomena. To the best of our knowledge, only two studies have examined this possibility. In Yamada et al. (2013, Experiment 2) morph sequences were generated that represented transitions between cartoon, stuffed, and real dogs. In Ferrey et al. (submitted, Experiment 1), bistable morph sequences were used that represented transitions between various non-human animals (e.g., between a duck and an elephant). In each of these studies, regions of maximal negative affect were found at intermediate levels of the perceptual continua, which is consistent with the generalized account of the UVH (see, Burleigh et al., 2013, Experiment 2). Between general formulations of the UVH and empirical support for UCV-like phenomena, greater theoretical consideration of the affective and cognitive processes is required to define the conditions under which the UCV will be observed as well as to differentiate it from related phenomena.

### Explanations for the uncanny valley phenomenon

Although the UVH provides a description of the non-linear response function, it does not explain why this function occurs, nor does it specify the mechanisms that are responsible. A common explanation is that the negative affect associated with uncanny stimuli might be a consequence of biological adaptations for threat avoidance behaviors (e.g., MacDorman et al., 2009). Stimuli within the valley might be convincing depictions of humans while falling short of a satisfactory level of human-likeness due to imperfections. These imperfections might cause them to be seen as “humans with disease” which triggers an aversive response (MacDorman and Ishiguro, 2006; MacDorman et al., 2009; Burleigh et al., 2013, Experiment 2). There is some evidence supporting this account. For example, Ho et al. (2008) observed that disgust could explain a significant portion of the variance in eeriness ratings. Furthermore, Steckenfinger and Ghazanfar (2009) observed that macaque monkeys displayed an aversion (as measured with looking times) to images of conspecifics that were of intermediate realism, which suggests that there might be an evolutionary basis to the phenomenon.

From this account, it might be reasonable to assume that the UCV phenomenon is specific to observers viewing images of conspecifics—an assumption that would be consistent with the classic UVH. Given that the spread of communicable diseases depends on the genetic similarity between the observer and the stimulus entity, it is possible that a species could have evolved mechanisms that allow them to respond to pathogen cues in conspecifics, but not heterospecifics. Communicable diseases, however, are not the only source of contamination that members of a species have had to contend with in their environments. As Rozin and Fallon (1987) point out, disgust is also a food-related emotion, which serves to prevent the oral incorporation of contaminated substances. As Schoenherr and Burleigh (2014) discuss, food substances that share features from two categories have been associated with aversive responses, such as food taboos (e.g., some refer to a certain transgenic tomato as a “Frankenfood” because it incorporates genes from a winter flounder). This suggests that the UCV phenomenon might not be specific to observers viewing images of conspecifics, but that it might also occur more generally in response to the categorical ambiguity of certain types of stimuli. Even if these accounts are correct, general learning mechanisms would also allow for the adjustment of diagnostic features of disease as well as inclusion and exclusion of categories associated with disease as a result of an individual's experiences with their environment.

Another theory that accounts for threat avoidance behavior is based on the premise that appearances provide information that allows individuals to predict behavior, and thus to anticipate potential threats in their environment. Some uncanny valley stimuli can be seen to present mismatched features (Seyama and Nagayama, 2007; MacDorman et al., 2009; Mitchell et al., 2011; Saygin et al., 2012), such as a machine with a convincingly human voice, or an android with a physical appearance that is highly realistic but movements that are robotic. In this account, stimulus features, such as physical appearances, drive the automatic selection of a neural model for the purpose of predicting behavior. Stimulus mismatches can therefore lead to the selection of an inaccurate neural model, which is associated with error-related brain activity (Saygin et al., 2012), and error-related processing might result in negative affect. These neural models thus require learning in order to acquire ontological categories that subsequently produce contrasts due to feature mismatch.

### The uncanny valley as categorical perception

If feature mismatch is the result of the inclusion of features from neighboring categories, then a crucial feature of any general account of the UVH is the specification of category learning systems that acquire the category structure, as well as the representations that are retained within them (for a recent review, see Goldstone et al., 2012). A number of studies have attempted to qualify the UVH by making reference to principles and processes associated with categorization generally, and categorical perception more specifically (Cheetham et al., 2011, 2013; Moore, 2012; Burleigh et al., 2013; Yamada et al., 2013; Ferrey et al. submitted). Categorical perception (CP) accounts of the UVH suggest that this phenomenon is a consequence of categorical processes associated with stimulus identification. Specifically, stimuli along a human-likeness continuum are perceived as members of either a “human” or “non-human” category, except at the category boundary where their membership is ambiguous. This follows from the position that stimuli at the category boundary should not provide the observer with sufficient perceptual evidence to allow easy or accurate identification on the basis of their representation of the category structure. As a consequence, uncertainty and negative affect are produced due to competition during categorization response selection (Cheetham et al., 2011; Burleigh et al., 2013), which might in turn activate conflict resolution processes like inhibitory devaluation (Ferrey et al., submitted).

Empirical evidence is consistent with accounts of the uncanny valley based on categorical perception. For instance, Cheetham et al. (2011, 2013) demonstrated that participants' response latencies were longest when categorizing stimuli that were located at, or adjacent to, the category boundary on a human-avatar morph continuum. In addition to this, Burleigh et al. (2013, Experiment 2), Ferrey et al. (submitted), and Yamada et al. (2013), have each observed non-linear affective response functions across between-category (including human-animal and animal-animal) morph sequences that peaked at the midpoint between categories where stimuli were most ambiguous. Relative to the categorization literature, these accounts are underspecified, and therefore do not provide a complete account of the UCV phenomenon. Moreover, whereas Cheetham et al. (2011, 2013) and Yamada et al. (2013) have made a crucial connection between categorization and the response patterns associated with the uncanny valley, we cannot assume that categorization performance will be the only, or even the primary, determinant of affect. As we discuss, the uncanny valley might also be attributed to sub-categorical processes, such as those involved in assessing stimulus frequencies (Zajonc, 1968; Bornstein, 1989).

### Category boundary and exemplar representations

Any explanation of the UCV phenomenon based on categorical perception must consider categorization processes and representational assumptions (e.g., prototype-related models were recently considered by Moore, 2012). Most CP accounts of the UVH appear to have assumed that categorization is governed by a “category boundary” representation (Cheetham et al., 2011, 2013; Burleigh et al., 2013). Category boundary models suggest that when a stimulus is encountered, it is used to locate and modify the location of a decision boundary in perceptual space (Ashby and Gott, 1988). When individuals are presented with a novel stimulus, they will compare its location in perceptual space to that of the category boundary. Proximity to the category boundary thereby increases categorization uncertainty (Paul et al., 2011; Schoenherr and Lacroix, 2014), and according to CP accounts of the UVH, proximity is also assumed to be inversely related to affect.

However, while a category boundary model might provide an adequate explanation of the uncanny “valley,” which is a simple U-shaped quadratic function, it cannot account for the entire UCV response function, which is a more complex N-shaped cubic function (e.g., Mori, 1970). We suggest that models that take into consideration exemplar-based information might account for the additional features of a more complex function. Exemplar-based models assume that a memory trace is encoded each time a stimulus is encountered (Medin and Schaffer, 1978; Nosofsky, 1984). During the course of learning, each instance becomes associated with a category label, and at the end of learning each exemplar is represented by a probability distribution of features. Over the course of learning, an individual's attentional focus becomes reweighted to different regions of the stimulus continuum (Nosofsky, 1984, 1986), such that attention is sensitized to between-category differences and desensitized to within-category differences. When presented with a novel exemplar, individuals will compare it to all exemplars available in memory, and the similarity between the new item and old items in memory will determine the new item's category membership.

Thus, a key difference between category boundary and exemplar-based models is how individuals become sensitized to perceptual space. Category boundary models suggest that individuals can only typically become sensitized to a single region of perceptual space, namely where the category boundary is located; whereas exemplar-based models suggest that individuals can become sensitized to multiple regions of perceptual space, due to the distributions of individual members (Nosofsky, 1984, 1986).

### The UCV as categorical perception or frequency-based exposure

Crucially, affective processing of stimuli might not require the instantiation of categorical processes. The mere-exposure effect (Zajonc, 1968) suggests that repeated exposure to stimuli can lead to the formation of preferences, and negative affect might therefore be accounted for on the basis of familiarity or perceptual fluency alone (for a review, see Bornstein, 1989). In support of this, Harmon-Jones and Allen (2001) reported physiological evidence (via EMG and EEG) of affective responses that resulted from mere-exposure to stimuli, which corresponded with self-reported evaluations. If the mere-exposure effect can be extended to all members of a perceptual continuum, then an observer's familiarity with individual members of the continuum might be able to explain non-linear affective response functions. For example, along a human-likeness continuum that is anchored by “human” and “robot,” individuals will have encountered a comparatively larger number of human instances relative to robots. Instances within these two categories should be much more familiar than instances that combine their features (e.g., androids). Thus, in contrast to the categorical perception account, a negative affective peak at an intermediate region in perceptual space might be explained by the fewer number of instances with the conjunction of features represented by stimuli in that region. On this basis, we suggest two distinct accounts of the UCV.

**Figure 2:**
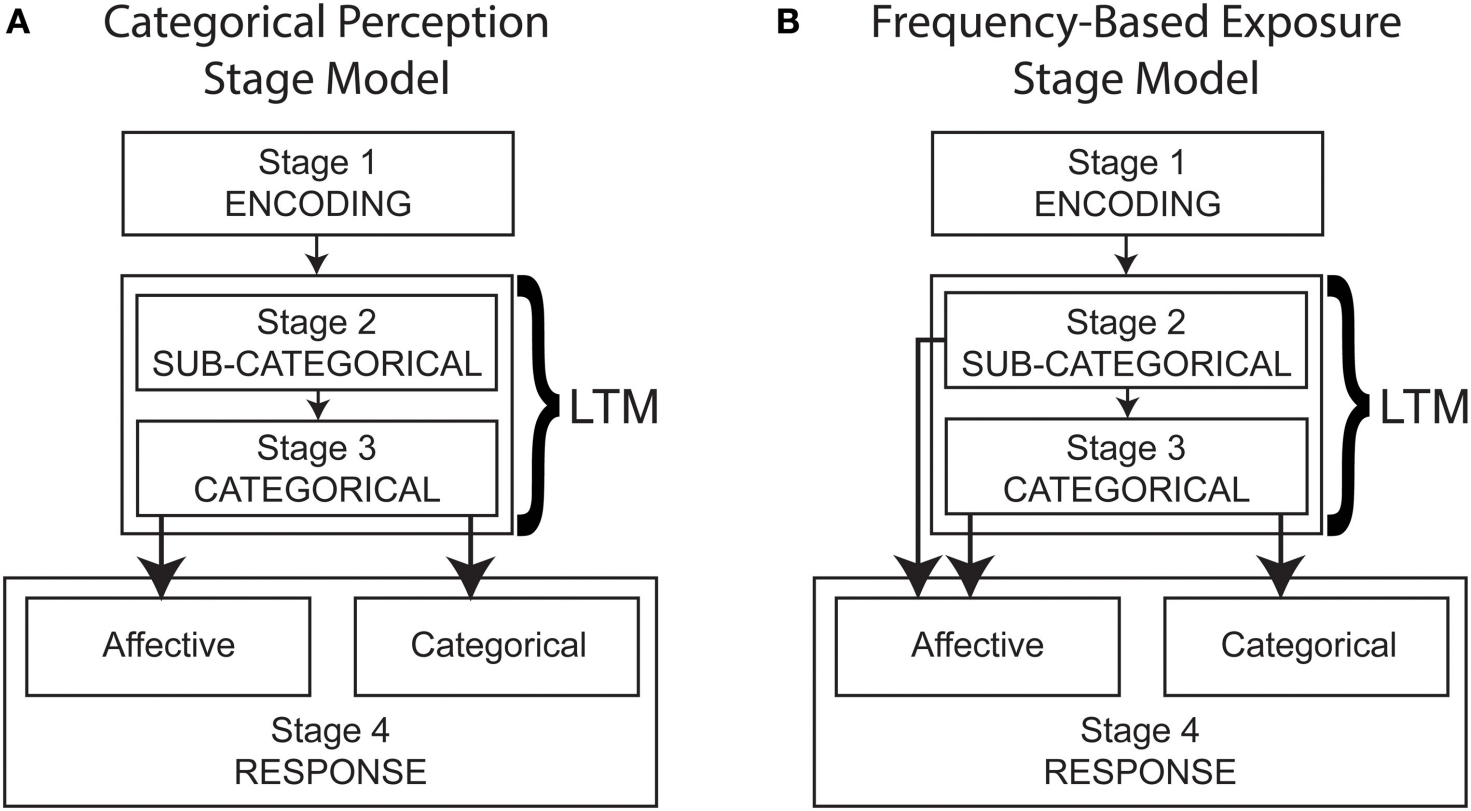
**Multistage UCV processing models derived from categorical perception (A) and frequency-based exposure accounts (B)**. Stage 1 (Encoding) relies on immediate memory whereas Stage 3 (Categorical) requires long-term memory (LTM) activation. Stage 2 (Sub-Categorical) requires activation of frequency-based properties retained in long-term memory.

We suggest that at least two broad relationships are possible between cognitive and affective processing of stimuli, which we conceptualize as categorical perception (Figure 2A) and frequency-based exposure (Figure 2B) stage models. In conceptualizing these models, we limit ourselves to unidirectional processing. We assume that stimulus processing is mediated by the information that is stored in long-term memory, which includes memory traces of past episodes.

The categorical perception model (Figure 2A) reflects our understanding of extant categorical perception accounts of the UCV, in that it assumes categorical and affective responses derive from a common processing stage. In this model, individuals process sub-categorical information such as basic perceptual properties (e.g., stimulus magnitude, orientation) and frequency, but this information does not directly influence responding. Subsequent to this stage, category structures stored in long-term memory are activated, and these structures are used to determine both affective and categorical responses.

Alternatively, the frequency-based exposure model (Figure 2B) assumes that categorical and affective responses derive from separable processing stages. Specifically, affective responses are also driven by sub-categorical processing, which relies on frequency-based memory representations to provide more basic information such as frequency. The models defined in Figures 2A,B are sufficiently distinct that their predictions can be tested in a category-learning paradigm.

### Present study

The present study was designed to test the predictions of the multistage models of the uncanny valley presented in Figures 2A,B, and a nested prediction concerning category structures. In the categorical perception model, the affective and categorical responses are derived from the same processing stage. Such a model therefore leads to a prediction of similar patterns of affective and categorical responses, as well as a strong and positive correlation between them. In contrast to this, the frequency-based exposure model implies that categorical and affective responses each account for unique sources of variance. Such a model therefore suggests that under some conditions patterns of responses might be similar, but they need not show a significant correlation.

Importantly, the stage models do not make predictions concerning the specific nature of categorical processing, only the relationship between categorical and affective responses. Therefore, a nested prediction concerns whether categorization will reflect category boundary or exemplar-based representations. The first possibility is that individuals will only have access to a category boundary representation that partitions the response continuum. Therefore, categorization accuracy and affective responses should increase, and response times should decrease, as a function of a stimulus' distance away from the category boundary. If participants are insensitive to individual characteristics, then categorization uncertainty should also be evidenced by a linear increase in response latencies as a function of proximity to the category boundary. Alternatively, if exemplar-based representations are acquired for two contrasting categories and used for categorical processing, then the location of the central tendencies for each category should determine the location of the maxima and minima of the response functions for affective and categorization responses. Response latencies should evidence a similar trend. Specifically, if uncertainty in category membership is a function of exemplar frequency, then we would expect exemplars presented with comparatively high frequency during training to be associated with fast responses whereas exemplars presented with comparatively low frequency to be associated with slow responses.

In order to test the predictions of these models, our experimental design uses a category-learning paradigm in which we manipulate exemplar frequency along the perceptual continuum. Experiment 1 consists of two training conditions. In the first condition, stimuli within response categories are presented with equal frequency, with each category having an equivalent distribution (EFED). In the second condition, both category distributions are equivalent, but the exemplars were presented with unequal frequency (UFED) such that stimuli near the extrema of Categories A and B training sets are presented with the greatest frequency, and stimuli adjacent to the category boundary were presented with the lowest frequency. An important aspect of our design is that individuals are not exposed to the continuum extrema during the training phase. Thus, while the category boundary of the EFED and the UFED conditions should be identical, differences in exemplar frequency should decrease affective responses outside the training range if frequency-based information is a determinant of categorical and/or affective responses.

The results of Experiment 1 should provide a straightforward tests of our predictions. Left unaddressed, however, is what we consider to be a tacit property of UCV as discussed by Mori (1970): we are presented with less exemplar variability within one category (e.g., human) and greater exemplar variability in the contrasting category (e.g., non-human). In Experiment 2, we used one category defined by exemplars with equal frequencies selected from the EFED condition and another category defined by exemplars with unequal frequencies selected from the UFED condition. This procedure resulted in an unequal frequency, unequal distribution condition (UFUD) which we take as a closer approximation to the properties of the UCV first proposed by Mori (1970). Table 1 provides training set frequencies.

**Table 1:** **Stimulus Frequencies for Training Session in Experimental Conditions for equal frequency, even distributions (EFED), unequal frequency, even distributions (UFED), and unequal frequency, uneven distributions (UFUD)**.

	**1**	**2**	**3**	**4**	**5**	**6**	**7**	**8**	**9**	**10**	**11**	**12**	**13**	**14**	**15**	**Tot**.
EFED	–	–	4	4	4	4	4	–	4	4	4	4	4	–	–	40
UFED	–	–	8	6	4	2	–	–	–	2	4	6	8	–	–	40
UFUD	–	–	8	6	4	2	–	–	4	4	4	4	4	–	–	40

Crucially, we were also interested in determining whether the affective response patterns could reasonably support a UCV interpretation. We distinguish between “strong” and “weak” interpretation as follows. The UCV function is a non-linear response function that is defined by a slope, indicating a category preference attributable to familiarity (e.g., for humans over robots), and a valley region that is located near the category boundary but skewed toward the preferred category. Thus, support for a strong interpretation of the UCV would be obtained if a response function possessed all of these features; support for a weak interpretation of the UCV would be obtained if a response function possessed some of these features, such as a valley region without a slope. We anticipate the possibility that the EFED and UFED conditions might provide support for a weak interpretation, but not for the strong interpretation, due to their symmetry. In contrast, the UFUD condition might provide support for a strong interpretation of the UCV due to the asymmetry of the response function.

Although the stimuli that we use all represent non-human entities, we believe the findings of these studies are pertinent to human-like stimuli. By using non-human stimuli we hope to minimize the influence of stimulus familiarity or preference for human stimuli. This novelty facilitates the task of training participants to learn different category structures in an experimental setting with practical limitations (e.g., time). This manipulation also allows us to illustrate that response patterns associated with the UCV are generally patterns that can be attributed to stimulus familiarity rather than human-likeness, *per se*.

## Experiment 1

Experiment 1 was designed as an initial test of our predictions derived from the hypothesized multistage models, and to provide evidence in support of the UCV phenomenon. We manipulated the frequency of stimulus presentation to differentially sensitize participants to regions of the stimulus response continuum. An equal frequency condition (EFED) was provided to half of the participants, wherein all stimuli within a category were presented with equal frequency, thereby creating a uniform distribution. An unequal frequency condition (UFED) was provided to the remaining half of the participants, wherein stimuli located within the middle of each category distribution were presented with higher frequency, thereby approximating a normal distribution. In each case, distributions of stimuli from Category A and Category B were symmetrical. Thus, by the end of training we hypothesized that participants should learn the distribution of the training stimuli equally well. Following training, participants responded to stimuli selected from the entire continuum. In the UFED condition, we additionally predicted that participants should show changes in affective responses due to less familiarity with the extreme values that in fact share fewer features with the contrasting category.

### Methods

#### Participants

A total of 60 participants were recruited online for this study (31 female, *M*_age_ = 37.2). Participants were recruited from Amazon's Mechanical Turk platform and paid a total of $5 if they completed all 4 sessions of the study ($1 for session 1, $1.25 for sessions 2 and 3, and $1.5 for session 4). All participants were registered with Mechanical Turk as United States residents. No participants reported having a visual impairment, and therefore no participants were excluded from our analyses. All participants consented to participate in the study.

#### Stimuli

Three morph sequences were generated, comprising the permutations of three distinct non-human creatures: a beast, a reptile, and an alien. These creatures were selected given our assumption that participants would have less familiarity with these categories thereby allowing us to more readily manipulate their frequency of exposure in the experimental context. Creatures were created using Daz Studio 4.6 Pro (daz3d.com) by modifying the morphology and texture of the *Genesis* base figure. Morph sequences were then created by stepwise adjustment of morphology and texture parameters corresponding to each creature. For example, the reptile creature had a “head scale” parameter which determined the size of its head, with a value of 32, whereas the alien creature had a value of 40. Therefore, the stimulus at the midpoint on the alien-reptile morph continuum assumed a value for this parameter that was half-way between the alien and reptile values (i.e., 36). Stimuli were then cropped in photo-editing software using an elliptical mask, and saved as images with a vertical resolution of 548 pixels. Stimuli were divided into training and test sets. The following stimuli were excluded from all training sets: stimulus 6 (the category boundary), and stimuli 1, 2, 14, and 15 (the extrema). Other stimuli were excluded depending on the frequency condition. For instance, stimuli 7 and 9 were not included in the training set for the UFED condition due to the frequency manipulation.

#### Procedure

**Figure 3:**
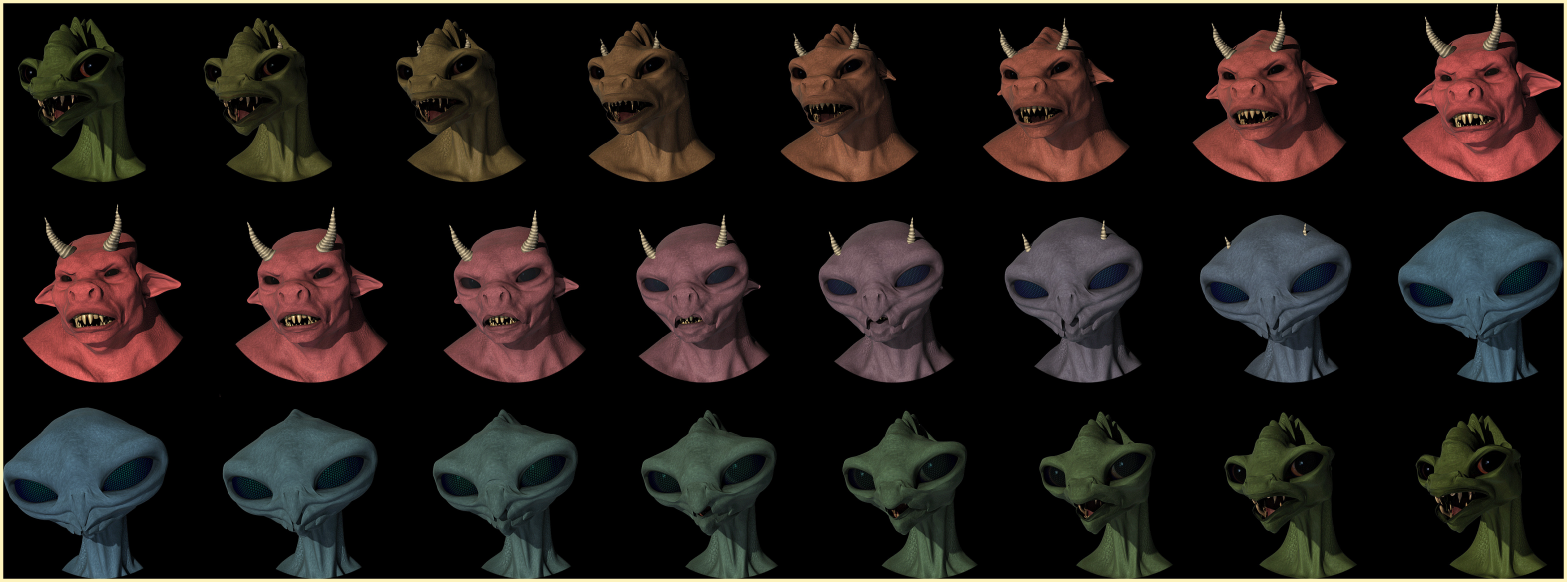
**Reptile-beast, beast-alien, and alien-reptile morph continua; stimuli shown here: 1, 3, 5, 7, 9, 11, 13, and 15**.

***Training***. At the start of the experiment, participants were presented with stimuli during the training and test phases of the experiment by randomly assigning them to a creature continuum (for an example, see Figure 3) and a frequency condition (see Table 1). In order to control for the effect of creature continua, we used a counter-balanced design such that an equal number of participants were assigned to each of the (creature x frequency) conditions. This resulted in a total of 5 participants for each cell of the design, or 30 participants in each of the experimental conditions that were of interest. In the EFED condition, participants received an equal presentation of stimuli selected from the training range, whereas in the UFED condition participants received an unequal presentation of stimuli selected from the training range; in each case the frequency distributions were symmetrical.

At the beginning of training, participants were instructed that they would be presented with “models of unfamiliar living creatures” and that their task was to “learn what categories they belonged to.” They were told that each creature was either a “Cax” or a “Miv” and that they were to press the “C” or “M” key depending on which type of creature they thought they saw. Participants were instructed to balance the demands of speed and accuracy. Key assignment was counter-balanced across participants.

Participants completed 1 training session per day over the course of 3 days. Each training session was composed of 10 blocks of 40 trials each, for a total of 400 trials per training session, and each session required approximately 20 min to complete. For each trial, a fixation point was presented for 500 ms, followed by a randomly selected stimulus from the training distribution for 750 ms (these timings were selected to be consistent with Cheetham et al., 2011). At the end of this sequence the response alternatives were presented until a response was registered. After a response was registered, feedback in the form of a “correct” or “incorrect” message was presented for 500 ms.

***Test***. In the test session, all 15 stimuli were presented. Unlike the previous blocks, we sought to limit the amount of exposure to previously unseen stimuli. Therefore, the test session consisted of 4 blocks, in which each stimulus was presented 2 times each, for a total of 120 trials. The training session required approximately 12 min to complete. Stimulus presentation preceded in the same manner as in the training phase with two notable exceptions. Following presentation of a stimulus, participants were asked to rate its eeriness on a scale ranging from 1 (not at all eerie) to 7 (extremely eerie) using the “1” through “7” keys, respectively. After registering their response, participants were then asked to indicate whether it was a “Cax” or “Miv,” as in previous sessions. The ordering of affective and categorization responses was deliberate in order to ensure that the effect of categorical information on ratings of eeriness would be limited.

***Implementation***. The study was developed for the web using HTML and JavaScript programming languages for the frontend, and PHP/MySQL for the backend. Preliminary tests using an automated responder on a test machine revealed that response time noise was within acceptable limits (i.e., less than 35 ms). Our online research environment is comparable to the one used by Crump et al. (2013). Crump et al., used JavaScript and recruited Mechanical Turk participants to successfully replicate numerous reaction time tasks like the Stroop (1935).

## Results

In order to test our predictions, we analyzed training and test responses separately in terms of categorization accuracy, response time, eeriness ratings, and the shape of categorization and affective response functions. A series of repeated-measures analyses of variance (ANOVA) were conducted. Greenhouse-Geisser adjusted values are reported with unadjusted degrees of freedom. All reported pairwise comparisons were conducted using a Bonferroni adjustment. We also report partial-eta squared as a measure of effect size. Following this, we use curve fitting analyses in order to facilitate our interpretation of the affective response functions.

### Training phase

#### Categorization accuracy

A repeated-measures ANOVA was conducted on categorization accuracy, using stimulus location relative to the category boundary (4) and response category (2) as within-subjects variables, and stimulus training distribution (2) as a between-subjects variable. Here, response categories (i.e., “Cax” and “Miv”) were randomly assigned to stimuli located on the left- and right-halves of the stimulus continuum. Given the counterbalancing of stimulus sets, we collapsed across morph models prior to analysis. Stimuli directly adjacent to the category boundary in the UFED training condition were also removed prior to analysis. This adjustment was made due to the fact that these stimuli were not present in the EFED training condition and might introduce bias in the analysis. Similarly, the stimulus located at the category boundary was not presented during training and was therefore absent from the analysis of test responses.

**Figure 4:**
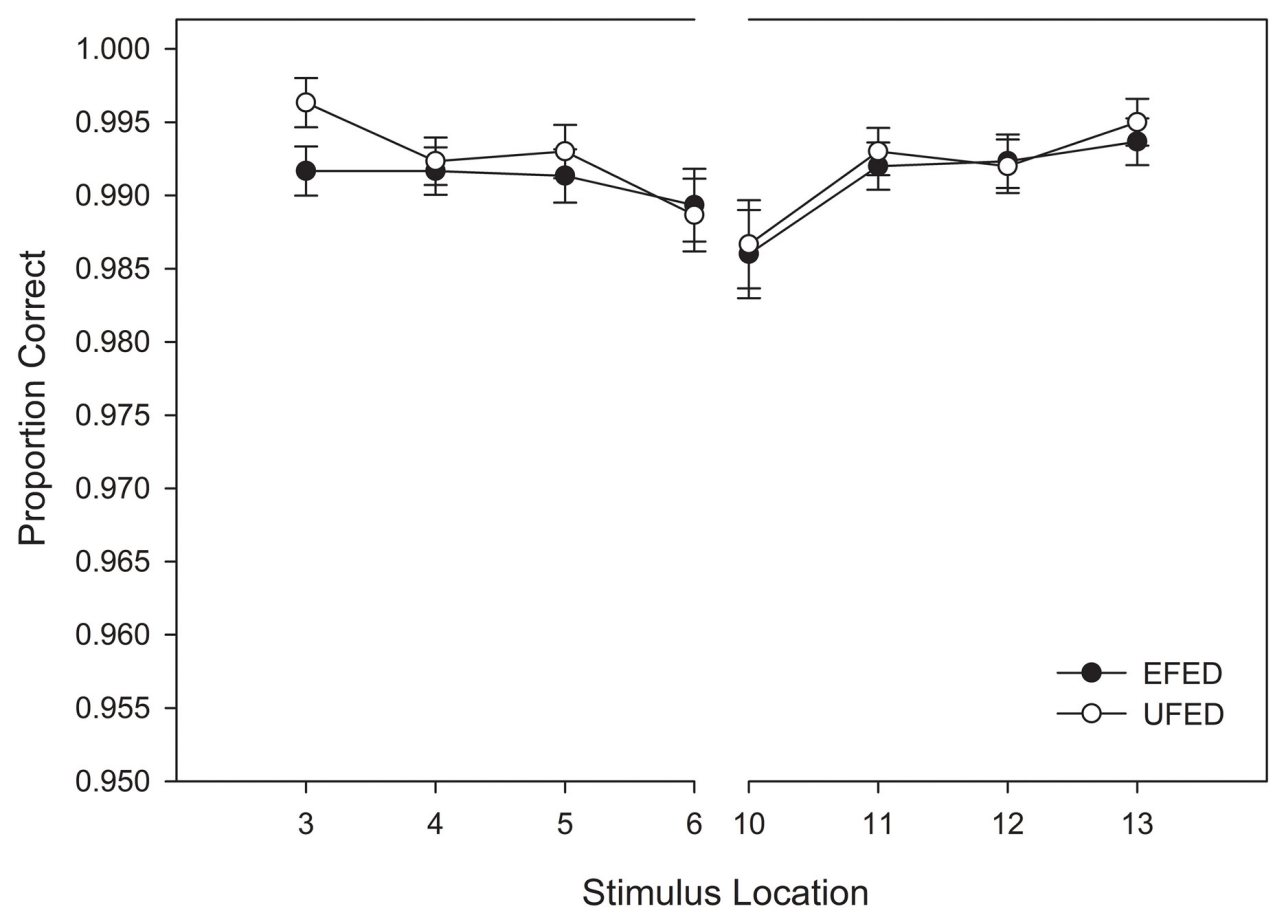
**Training categorization response accuracy in equal frequency (EFED) and unequal frequency conditions (UFED)**. Stimulus values correspond to stimuli selected from the training range (i.e., stimuli 3–13). Error bars represent 1 standard error of the mean *N* = 60).

Our analysis of training response accuracy revealed a significant main effect for stimulus distance from the category boundary, *F*_(3, 174)_ = 11.921, *MSE* < 0.001, *p* < 0.001, η^2^_*p*_ = 0.17. As Figure 4 suggests, categorization accuracy increased as a function of distance from the category boundary with the lowest accuracy observed for stimuli nearest the category boundary (Stimuli 6 and 10) and the greatest accuracy observed for the most extreme stimuli (Stimuli 3 and 13). Supporting our interpretation of the data, pairwise comparisons revealed significant differences between stimuli nearest the category boundary (i.e., stimuli 6 and 10) and stimuli at all other distances (*p*s < 0.012). No other main effects or interactions reached significance, *p*s > 0.1^1^. Thus, the primary determinant of categorization accuracy during training was the location of a stimulus along the morphed continuum.

#### Categorization response times

Using the same design as the analysis of accuracy, a repeated-measures ANOVA was conducted on categorization response time. To eliminate outlying observations, we first computed an unadjusted mean response time for each participant and identified trials wherein their responses were 3 standard deviations above the mean. This accounted for 2.1% of trials. Consistent with our analysis of categorization accuracy, categorization response time decreased as a function of distance from the category boundary, *F*_(3, 174)_ = 5.061, *MSE* = 1380.276, *p* = 0.005, η^2^_*p*_ = 0.08.

**Figure 5:**
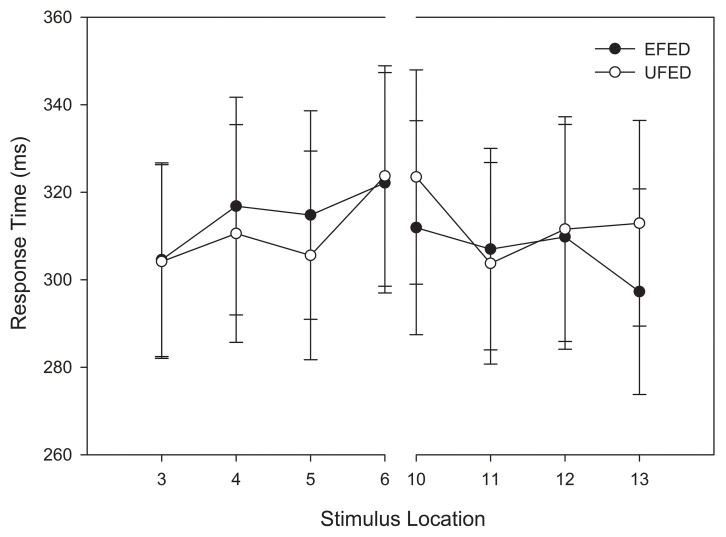
**Training categorization response time in equal frequency (EFED) and unequal frequency conditions (UFED)**. Stimulus values correspond to stimuli selected from the training range (i.e., stimuli 3–13). Error bars represent 1 standard error of the mean (*N* = 60).

As Figure 5 demonstrates, stimuli near the category boundary were associated with longer response times than stimuli at more distal locations. Pairwise comparisons revealed that the response latency for stimuli close to the category boundary significantly differed for adjacent stimuli and those located at extreme distances (*p*s < 0.044). No other main effects or interactions reached significance, *p*s > 0.1. Again, these results provide additional evidence that the primary determinant of categorization response time during training was the location of a stimulus along the morphed continuum.

### Test phase

#### Response accuracy

A repeated-measures ANOVA was conducted on accuracy of responses obtained during the test phase. This analysis was comparable to that of the training phase with the exception that due to the uniform distribution used during the test phase for both training groups, stimuli adjacent to the category boundary as well as novel extrapolation items outside the range of the training items were also included in the analysis. Again, the stimulus located at the category boundary was eliminated from the analysis due to its ambiguity (it was entered into another analysis, see the Categorization response times and Affective ratings of eeriness sections below). Thus, stimulus location relative to the category boundary (7) and response category (2) were entered as within-subjects variables, and stimulus training distribution (2) was entered as a between-subjects variable.

**Figure 6:**
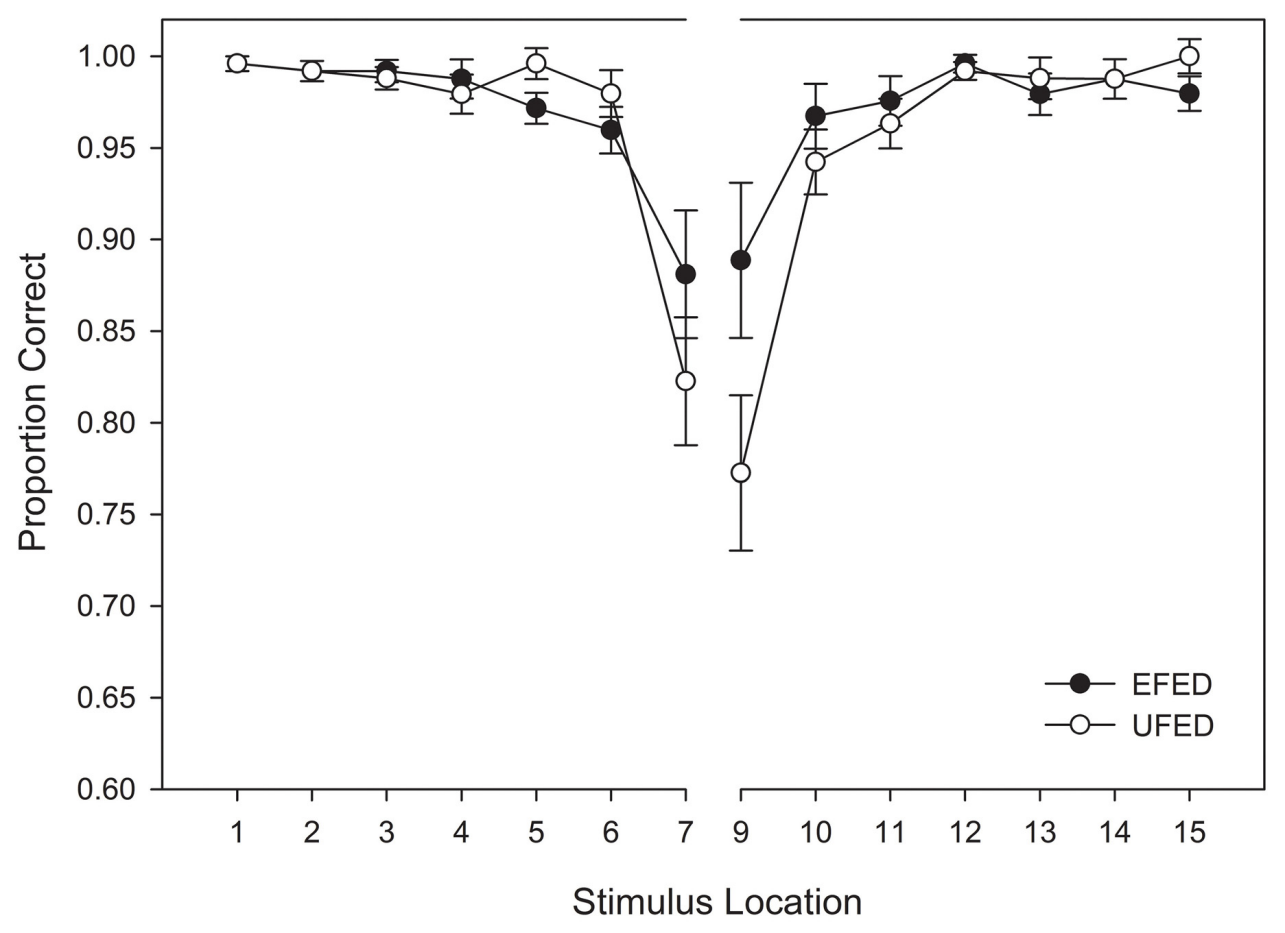
**Test categorization response accuracy in equal frequency (EFED) and unequal frequency conditions (UFED)**. Stimulus values correspond to stimuli selected from the entire stimulus range (i.e., stimuli 1–15). Error bars represent 1 standard error of the mean (*N* = 60).

Replicating the findings of categorization accuracy obtained in the training phase, we observed a significant main effect of stimulus distance from the category boundary, *F*_(6, 348)_ = 54.516, *MSE* = 0.021, *p* <0.001, η^2^_*p*_ = 0.485. An interaction was also observed between stimulus distance and frequency distribution, *F*_(6, 348)_ = 5.292, MSE = 0.021, *p* =0.007, η^2^_*p*_ =0.08^2^. An examination of Figure 6 reveals a more pronounced decrement in categorization accuracy around the category boundary in the test phase relative to the training phase. This trend was especially pronounced for participants in the unequal frequency (UFED) training condition, and suggests that participants were affected by the distributional properties in the test phase.

#### Categorization response times

A repeated-measures ANOVA was conducted on categorization response time in the test phase. In order to compare to affective response functions (see below), the stimulus at the category boundary was also included. Therefore, unlike previous analyses, the entire stimulus continuum was tested. Thus, stimulus location (15) was entered as a within-subjects variable and stimulus training distribution (2) as a between-subjects variable. An analysis of response time outliers was again conducted on individual participants' responses. After obtaining an unadjusted mean, no responses were observed to be larger than 3 standard deviations above the mean. This result is not surprising given the reduced number of replications in the test phase.

**Figure 7:**
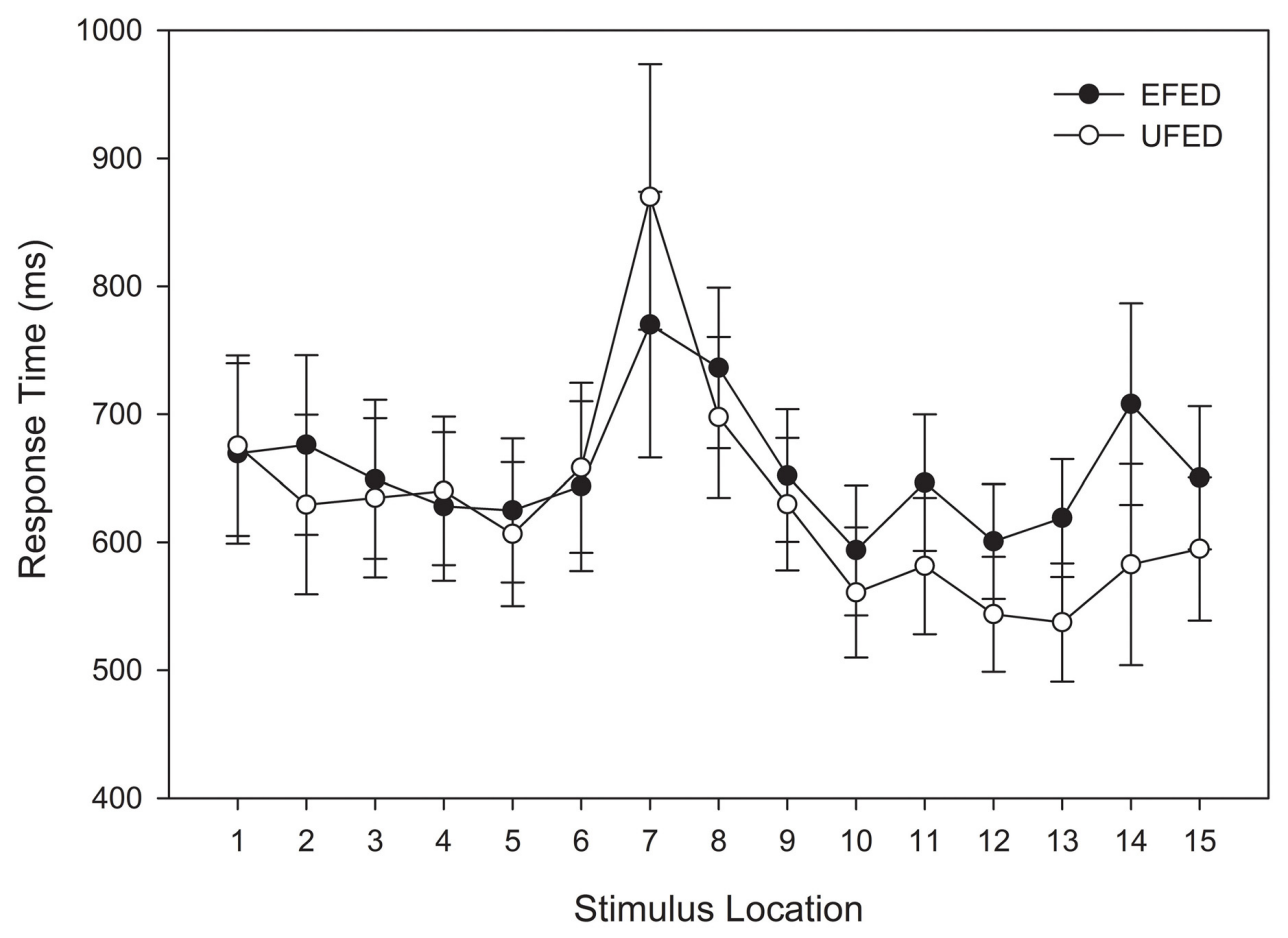
**Test categorization response time in equal frequency (EFED) and unequal frequency conditions (UFED)**. Stimulus values correspond to stimuli selected from the entire stimulus range (i.e., stimuli 1–15). Error bars represent 1 standard error of the mean (*N* = 60).

As with the response time analysis in the training phase, a main effect was observed for stimulus location, *F*_(14, 812)_ = 4.631, *MSE* = 188391.325, *p* = 0.002, η^2^_*p*_ = 0.053. Figure 7 indicates that this effect can be accounted for by the slower response times for stimuli that were at, and adjacent to, the category boundary.

#### Category response frequencies

In the categorical perception literature, category boundaries were originally assessed by examining category response frequencies across a stimulus continuum (cf. Pisoni and Tash, 1974). These analyses allow for the identification of the location and shape of a category boundary. Whereas continuous increases in stimulus magnitude relative to some criterion (e.g., brightness, size) can be fit a continuous function, categorical perception is typically reflected in a sigmoidal function (Harnad, 1987). Our analyses determined the frequency of “Cax” responses as a proportion of total category responses for each level along the stimulus continuum (e.g., frequency of “Miv” responses reflects the inverse of this function). These results were then plotted across the stimulus continuum, and a sigmoid function was fitted to the data.

**Figure 8:**
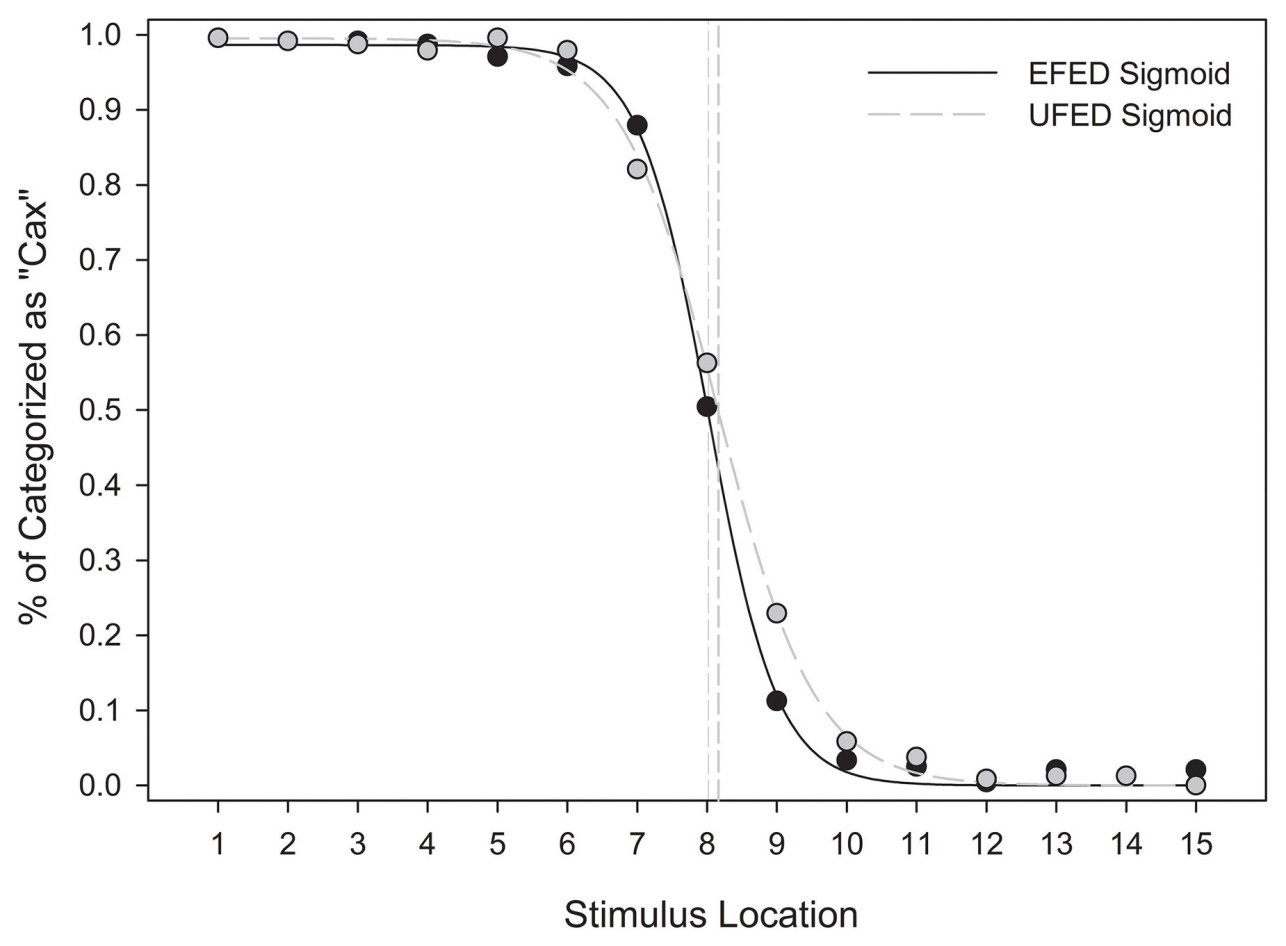
**Mean categorization response frequencies for equal frequency (EFED) and unequal frequency conditions (UFED)**. Stimulus values correspond to stimuli selected from the entire stimulus range (i.e., stimuli 1–15). Vertical dashed lines represent the estimated category boundaries.

Figure 8 suggests that the category response frequencies in EFED and UFED conditions were consistent with a sigmoidal shape, and indicate that a category boundary was present at or near stimulus 8, the mid-point of the stimulus continuum. The sigmoid function provided an adequate fit in the EFED [*F*_(2, 12)_ = 7943.437, *MSE* < 0.001, *p* < 0.001, *R*^2^_*adj*_ > 0.999] and UFED [*F*_(2, 12)_ = 8110.364, *MSE* < 0.001, *p* < 0.001, *R*^2^_*adj*_ > 0.999] conditions. Parameter estimates confirm that the point of inflection in each case was approximately located at stimulus 8 (x_0,EFED_ = 8.020, x_0,UFED_ = 8.165). Thus, stimulus identification in the test phase is consistent with categorical perception.

#### Affective ratings of eeriness

In order to examine the location and property of the global minima in eeriness ratings that correspond to the uncanny valley, we conducted a repeated-measures ANOVA that included stimulus location (15) as a within-subjects variable and stimulus training distribution (2) as a between-subjects variable. Relative to the previous analyses of categorization accuracy and response time, this approach allows for a straightforward comparison between the shapes of the function that fit affective responses provided below. Our analysis revealed a marginally significant effect of stimulus location, *F*_(14, 812)_ = 3.267, *MSE* = 15.128, *p* = 0.055, η^2^_*p*_ = 0.053. The interaction between stimulus location and training distribution did not approach significance, *F*_(14, 812)_ = 1.706, *MSE* = 15.128, *p* = 0.193, η^2^_*p*_ = 0.029.

**Figure 9:**
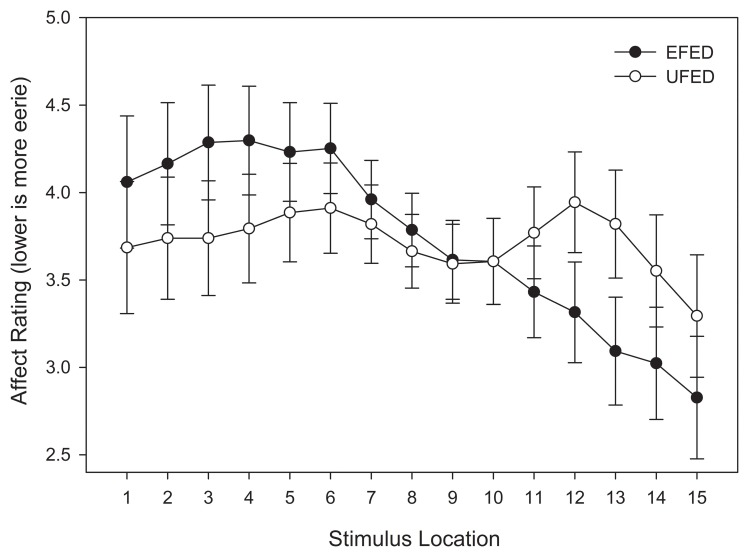
**Test affective responses (lower is more eerie) in equal frequency (EFED) and unequal frequency conditions (UFED)**. Stimulus values correspond to stimuli selected from the entire stimulus range (i.e., stimuli 1–15). Error bars represent 1 standard error of the mean (*N* = 60).

Although an interaction was not observed between stimulus location and stimulus training distribution, Figure 9 suggests that the trend of affective responses did change as a function of stimulus training distribution. In the EFED condition, an overall linear trend was observed across the continuum, with an affective minimum at one end of the stimulus continuum, and an affective maximum at the other end. Such a pattern would be expected if participants were using one response category as reference point and comparing stimulus exemplars to that category. By contrast, in the UFED condition an M-shape was instead observed, with affective minima at the category boundary as well as at the end-points of the stimulus continuum. When comparing these results to those obtained in the analyses of categorization responses, it is instructive to note that the stimuli associated with the slowest response times and lowest levels of categorization accuracy were not those that generated the highest levels of eeriness. As such, it is reasonable to conclude that there is a degree of dissociation between categorization performance and affective responses. Thus, while categorical perception appears to be compatible with the uncanny valley hypothesis, it also appears that the affective component of the valley is influenced by other affective and cognitive processes.

#### Correlations

The variation in patterns across our dependent measures prompted an examination of the relationship between these measures. Test accuracy, test response time, and affective ratings were included in a correlational analysis. We examined the EFED and UFED conditions separately.

As Table 2 indicates, in both the EFED and UFED conditions, marginally significant negative correlations were obtained between response time and categorization accuracy, *r*_(14)_ = −0.485, *p* = 0.079, and *r*_(14)_ = −0.517, *p* = 0.059, respectively. However, in both conditions, the correlation between categorization response time and eeriness did not reach significance, *p*s > 0.5. Thus, while an increase in response time was observed near the category boundary, the remaining differences in responses did not support an interpretation that response time and eeriness ratings were produced by the same response processes. Equally important, in both conditions, the correlation between accuracy and eeriness did not reach significance, *p*s > 0.5. Thus, while it appears that information processing associated with the production of a categorization response and affect were related, category membership and affective responses differed in important ways. These differences appear to be a result of novel extrapolation items, something that is inconsistent with a category boundary model of the UCV.

**Table 2:** **Pearson correlations of dependent measure in the test phase for equal frequency (EFED) and unequal frequency conditions (UFED)**.

		**Response Time**	**Accuracy**
**EFED**			
	Accuracy	−0.485 (0.079)	-
	Eeriness	0.076 (0.796)	−0.059 (0.842)
**UFED**			
	Accuracy	−0.517 (0.059)	-
	Eeriness	0.136 (0.643)	0.076 (0.797)

*p-values are in brackets*.

#### Curve fitting analysis

Mori's original proposal assumed that the uncanny valley is characterized by a non-linear response function. In the present experiment, we sought to directly test this assumption by fitting curves to the obtained response functions (see also Burleigh et al., 2013) for both EFED and UFED training conditions. A second goal of the present analysis was to obtain evidence for the underlying representation that supports the uncanny valley either in terms of a category boundary or an exemplar-based representation.

A number of non-linear functions were selected on theoretical grounds that were not included in Mori's original characterization of the model. In particular, our manipulation of frequency effects in the context of a categorization experiment was motivated by the belief that when participants are sensitized to specific regions of the response continuum, the location of affective minima and maxima can be manipulated. As we noted, a category boundary representation would be evidenced by a U-shaped quadratic function, whereas an exemplar-based category representation would be evidenced by an M-shaped quintic function.

We used a curve fitting analytic approach to test these possibilities, by fitting polynomials of degree 0 through 5 (i.e., constant, linear, quadratic, cubic, quartic, and quintic) to the means. Curve fitting was performed using Origin Lab (originlab.com) software. We used the Akaike Information Criterion (AIC; see Burnham and Anderson, 2002) as our goodness-of-fit index. The AIC is suited to comparing models with different degrees of complexity because it penalizes models with additional fit parameters. We calculated raw Akaike values and Akaike Weights (*w*_i_), which are a transformation of raw scores that indicate the probability that a particular model among the set of models is correct (Wagenmakers and Farrell, 2004). Using these weights, we also calculated evidence ratios by dividing the weight of one model by the sum of all weights. These ratios are understood in context of a “confidence set,” which is similar to a confidence interval and is defined as 10% of the highest Akaike Weight in the set (Royall, 1997). Thus, models falling outside of the confidence set can be rejected as poorer fits to the data. For the purposes of interpretation, it should be noted that lower raw Akaike values and higher Akaike Weights indicate a better fit to the data. The results of these analyses are summarized in Table 3.

**Table 3:** **Residual sums of squares (RSS) and Akaike values for equal frequency, equal distribution (EFED), unequal frequency conditions, equal distribution (UFED), and unequal frequency, unequal distribution (UFUD) conditions**.

**Training Set**	**Model**	***RSS***	***AICc***	***Δ_i_*(AIC)**	***w_i_*(AIC)**	***CI***
EFED	Constant^1^	3.521	−19.43	52.81	<0.001	0.054
Condition	Linear^2^	0.431	−48.26	23.98	<0.001	–
	Quadratic^3^	0.152	−60.70	11.54	0.002	–
	Cubic^4^	0.057	−71.61	0.63	0.396	–
	Quartic^5^	0.040	−72.24	0.00	0.542	–
	Quintic^6^	0.036	−67.84	4.40	0.060	–
UFED	Constant^1^	0.386	−52.59	4.76	0.064	0.069
Condition	Linear^2^	0.328	−52.36	4.99	0.057	–
	Quadratic^3^	0.246	−53.45	3.90	0.099	–
	Cubic^4^	0.235	−50.36	6.99	0.021	–
	Quartic^5^	0.135	−54.01	3.34	0.131	–
	Quintic^6^	0.073	−57.35	0.00	0.693	–
UFUD	Constant^1^	1.145	−36.28	23.26	<0.001	0.069
Condition	Linear^2^	0.534	−45.03	14.51	<0.001	–
	Quadratic^3^	0.188	−57.52	2.02	0.252	–
	Cubic^4^	0.181	−54.22	5.32	0.049	–
	Quartic^5^	0.093	−59.54	0.00	0.693	–
	Quintic^6^	0.091	−53.99	5.54	0.043	–

*Superscript denotes K, the number of parameters in the model*.

In the EFED condition, the constant, linear, and quadratic models fell outside the confidence set. Thus, accounts based on random responding or based on the association of equivalent negative affect for each stimuli are not supported (constant). Participants also did not appear to be solely biased by one end of the response continuum (linear). Perhaps most importantly for our purposes, a failure to obtain a fit for the quadratic function suggests that a category boundary model does not provide a good fit to the data in the absence of other assumptions. Instead, the model within the confidence set that was most likely to represent the data was the quartic model, *w*_i_(*AIC*) = 0.542. Thus, there is a 54.2% chance that the quartic model best accounts for the pattern or data we observed. However, the cubic model obtained an Akaike weight of a similar magnitude, *w*_i_(*AIC*) = 0.396. Given the similarity of these model weights, it would be reasonable to select the cubic function over the quartic function on the basis of its parsimony. This finding suggests that, for at least one category, the stimulus frequency manipulation produced a change in response affect and that a category boundary model cannot adequately account for the data.

In the UFED condition, we again observed that the constant and linear models fell outside the confidence set. In the same manner as the EFED condition, this suggests that a constant response bias or uniform negative affect were not evidenced in participants' responses (constant) and that a single category was not used as the sole basis for comparison (linear). Instead, the model within the confidence set that is most likely to represent the data was the quintic model. *w*_i_(*AIC*) = 0.693, and the next best model was the quartic model, *w*_i_(*AIC*) = 0.131. Thus, the obtained difference between these models clearly suggests that a quintic function best represents this data set. Taken along with the EFED results, the observation that a quintic function provides the best fit again suggests that the inclusion of exemplar-based representation is an important feature of a model of the UCV phenomenon.

## Discussion

The results of Experiment 1 add further evidence to the literature for the existence of the UCV phenomenon. Our results, however, qualify categorical perception accounts. Our measures of categorization accuracy, response frequencies, and response latencies all produced categorical response functions indicating that participants successfully learned the category structures. These analyses also suggest that the equivalent categorization performance was obtained on either side of the category boundary, which indicates that categories (i.e., Cax and Miv) were learned equally well. Similarly, whether participants were trained with exemplars with equal frequencies or unequal frequencies did not appear to alter the location of the category boundary. In both equal frequency (EFED) and unequal conditions (UFED), the category boundary was located at stimulus 8. Greater accuracy was obtained for items adjacent to the category boundary in the equal frequency condition relative to those in the unequal frequency condition. While such findings indicate categorical perception, it is not necessarily the case that categorical processing is the primary determinant of affective responses.

In order for the UCV to be understood in a manner similar to Mori's initial conceptualization, an affective relationship must be established with the location of exemplars along a continuum. Our curve-fitting analysis of eeriness ratings indicated that there were differences between the frequency training conditions. In the equal frequency condition, the response pattern was best fit by a cubic function. This pattern was evidenced by a slope, indicating that one response category was preferred to the other, and also a non-linear component at one end of the stimulus continuum. This pattern did not conform to our *a priori* hypotheses. Therefore, we can only speculate about its causes. One possibility is that the response pattern was an artifact of the category-response-key mappings that were used in our design. Each response category was assigned to a specific key on the keyboard, and participants were instructed to use index fingers on different hands for each key. As the location of the “C” and “M” keys are fixed on a standard QWERTY keyboard, handedness could have played a role. A second possibility is that the response labels themselves could have introduced some bias. For instance, participants might have preferred “Cax” because it occurs earlier in the alphabet, or because it was more familiar to them (due to associations with phonetically similar words), or they might have adopted a related response heuristic where in one category was used to anchor judgments (e.g., due to reading labels from left to right), or one of the category labels might have been more meaningful than another which resulted in differential leaning outcomes (as has been observed with non-sense syllables, see Davis, 1930). If these factors systematically affected performance, they do not appear to be evidenced in the unequal frequency condition. The asymmetries obtained in the EFED condition are likely a result of idiosyncratic response biases and strategies used by the participants in this condition. In the unequal frequency condition, the response pattern was best fit by a quintic function. This pattern was M-shaped, with a valley-region located near the category boundary, and two affective minima located near the extrema. Importantly, however, because the pattern was symmetrically distributed around the category boundary, it did not possess all of the features of the classic UCV function and therefore would only support a weak UCV interpretation.

The differences in response functions for categorization accuracy and eeriness ratings also suggest an important relationship that has been neither specified nor explicitly examined in the literature examining the UVH. Namely, we found that categorization accuracy and affect did not significantly co-vary. Such a finding has important implications for studies of the UVH that claim that it can be accounted for by categorical perception. We suggest that the methods of Experiment 1 played a key role in dissociating affective and categorical responses. By requiring affective responses immediately after stimulus presentation and prior to categorical responses, the probability that categorical information was available was reduced. Two differences in findings provide clear demonstrations of this dissociation. First, the small amount of error variance in the categorization responses observed for the items on the end of the distribution in the test session can be sharply contrasted against the larger error variance for the affective responses. Second, whereas categorization accuracy was uniformly high and response times were uniformly fast for items located near the ends of the distributions, affective ratings instead showed asymmetric effects with the response functions.

Another interesting finding was the absence of a relationship between response time and eeriness. Long response latencies are typically taken as evidence of response uncertainty. If eeriness is a consequence of uncertainty in the category membership of an exemplar, then eeriness and response times should exhibit a positive correlation. Instead, the absence of a significant correlation suggests that the processes that determine the uncertainty in category membership and the processes underlying eeriness might be supported by different affective and cognitive processes. Thus, whereas the uncanny valley appears to be a product of experience with exemplars, these two processes appear to be separable. It is necessarily the case that at some level of processing these processes must be influenced by the same stimulus information. Yet stages of processing appear to be evidenced such that affective ratings were influenced more by novel exemplars that represented extrapolations for the range of training stimuli, whereas categorization responses appear to be primary influenced by categorical representations of the stimulus continuum stored in long-term memory.

Experiment 1 therefore provides preliminary evidence in support of the frequency-based exposure model of the UVH, and against the categorical perception model. However, categorization responses were consistent with a category boundary representation. Therefore, we suggest that frequency-based memory representations and category boundary representations are both stored in long-term memory, but that the representation of this information produces different patterns of performance in affective and categorical responses. A remaining possibility is that the UCV phenomenon requires both unequal frequencies within a category, and unequal distributions for both reference categories. Experiment 2 examines this possibility.

## Experiment 2

Experiment 2 was conducted to clarify the relationship between variables observed in Experiment 1 while also further investigating the effect of distributional properties on categorical and affective responses. One way to interpret Mori's (1970) proposal is that there is nothing intrinsically important about the human category. Rather, it is only our frequency of exposure to, or familiarity with, stimuli that results in a category being used as a point of reference. As a result, non-human categories contrasted against the human category are likely to be perceived as less familiar due to their lower frequency. Thus, Mori's proposal might take for granted that two conditions need to be met for the experience of eeriness to occur when contrasting categories: a small number of items from one category need to be observed with high frequency and a larger number of items need to be observed from a contrasting category with unequal frequency.

### Methods

#### Participants

A total of 30 participants were recruited online for this study (12 female, *M*_age_ = 34.3). As before, participants were recruited from Amazon's Mechanical Turk platform and paid a total of $5 if they completed all 4 sessions of the study. All participants were registered with Mechanical Turk as United States residents. No participants reported having a visual impairment, and therefore no participants were excluded from our analyses. All participants consented to participate in the study.

#### Stimuli

The same stimuli were used to ensure a direct comparison with Experiment 1.

#### Procedure

All procedures were identical to Experiment 1 with the exception of training frequency. In Experiment 2, we presented participants with one category from the equal frequency condition in Experiment 1 and another category from the unequal frequency condition. We refer to this distribution as the unequal frequency, unequal distribution (UFED) condition below.

## Results

As in Experiment 1, we analyzed categorization accuracy, categorization response times, categorization response frequencies, and affective responses in the training and test phases. We also conducted curve fitting analyses to facilitate our interpretation of the affective response pattern. Again we report Greenhouse-Geisser unadjusted values and unadjusted degrees of freedom.

### Training phase

#### Response accuracy

A repeated measures ANOVA was performed on categorization accuracy, using stimulus location relative to the category boundary (4) and response category (2) as within-subjects variables. Unequal and equal frequency categories were assigned to stimuli located on the left- and right-halves of the stimulus continuum, respectively. Similarly, we also collapsed across morph models and excluded the category boundary stimulus, as well as stimuli directly adjacent to the category boundary, because training for these stimuli occurred for only one of the two response categories. As in Experiment 1, we obtained a significant main effect for stimulus distance from the category boundary, *F*_(3, 87)_ = 21.725, *MSE* < 0.001, *p* < 0.001, η^2^_*p*_ = 0.428. However, unlike Experiment 1, we also obtained a significant main effect for category, *F*_(1, 29)_ = 8.942, *MSE* < 0.001, *p* = 0.006, η^2^_*p*_ = 0.236, and a significant interaction between category and stimulus distance, *F*_(3, 87)_ = 20.951, *MSE* < 0.001, *p* < 0.001, η^2^_*p*_ = 0.419^3^.

**Figure 10:**
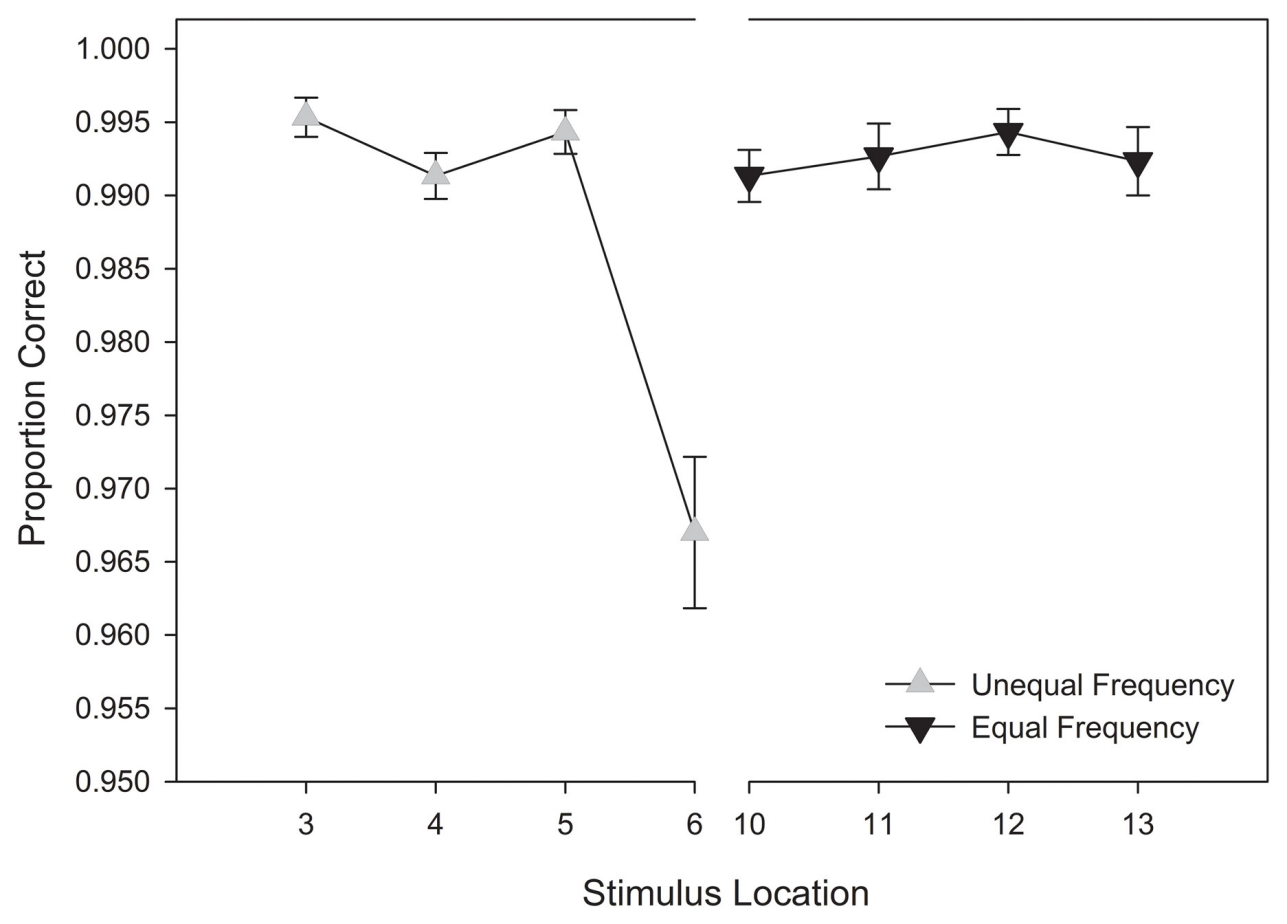
**Training categorization response accuracy in the unequal frequency, unequal distribution condition**. Stimulus values correspond to stimuli selected from the training range (i.e., stimuli 3–13). Error bars represent 1 standard error of the mean (*N* = 60).

Figure 10 suggests that this interaction can be accounted for by an asymmetry in the frequency of exemplars contained within the response categories. Specifically, with responses to the unequal frequency category, the stimulus nearest the category boundary (stimulus 6) was associated with lower categorization accuracy than stimuli at more distal locations. In contrast, the stimulus nearest the boundary (stimulus 10) in the equal frequency category received accuracy similar to other stimuli in its response category. These results replicate the unequal and equal frequency conditions in Experiment 1, respectively.

#### Response times

A similar repeated measures ANOVA was conducted for categorization response time. Prior to this analysis, we removed outlying trials with response times greater than 3 standard deviations from a participant's mean response time for each stimulus. This resulted in a removal of 2% of all trials. As with the analysis of accuracy, main effects were observed for exemplar distance from the category boundary, *F*_(3, 87)_ = 9.048, *MSE* = 780.019, *p* < 0.001, and response category, *F*_(1, 29)_ = 6.481, *MSE* = 635.357, *p* = 0.016, as well as an interaction between stimulus distance and response category, *F*_(3, 87)_ = 13.83, *MSE* = 538.695, *p* < 0.001.

**Figure 11:**
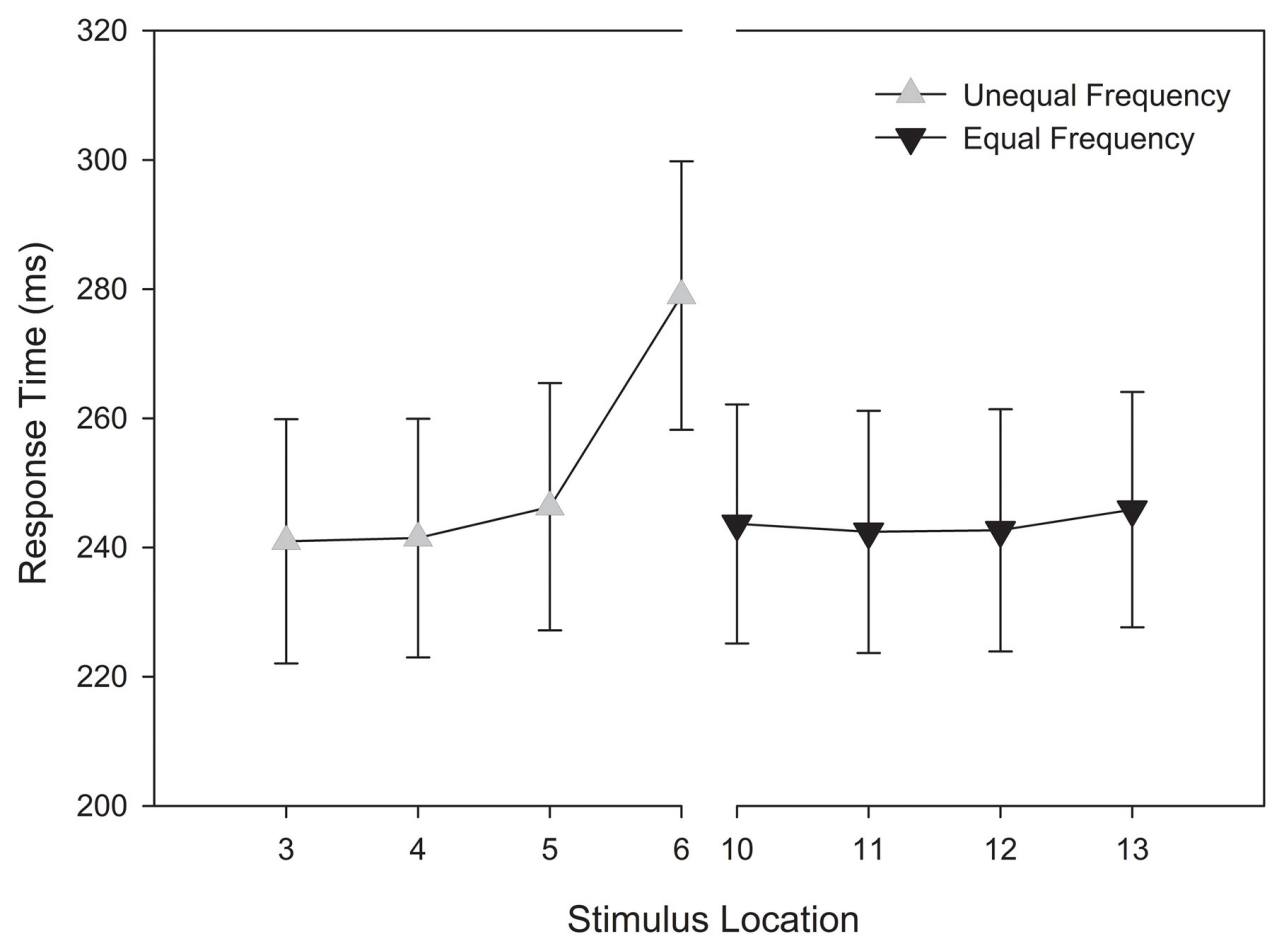
**Training categorization response time in the unequal frequency, unequal distribution condition**. Stimulus values correspond to stimuli selected from the training range (i.e., stimuli 3–13). Error bars represent 1 standard error of the mean (*N* = 60).

Figure 11 suggests that, as in the case of accuracy, this interaction can be accounted for by an asymmetry in the response categories. Specifically, in the unequal frequency condition, the stimulus nearest the category boundary (stimulus 6) was associated with slower response times than were stimuli at more distal locations. In contrast, stimulus 10 in the equal frequency condition was associated with response times equivalent to those of other stimuli in its response category.

### Test phase

#### Response accuracy

A repeated measures ANOVA was also conducted on categorization response accuracy for stimuli presented during the test phase. As in Experiment 1, this analysis included stimuli adjacent to the category boundary, because participants received an equal frequency of these stimuli in each response category during test. Again, the category boundary stimulus was not included, as there was no objective criteria that could be used to determine accuracy. Thus, stimulus location relative to the category boundary (7) and response category (2) were entered as within-subjects variables.

**Figure 12:**
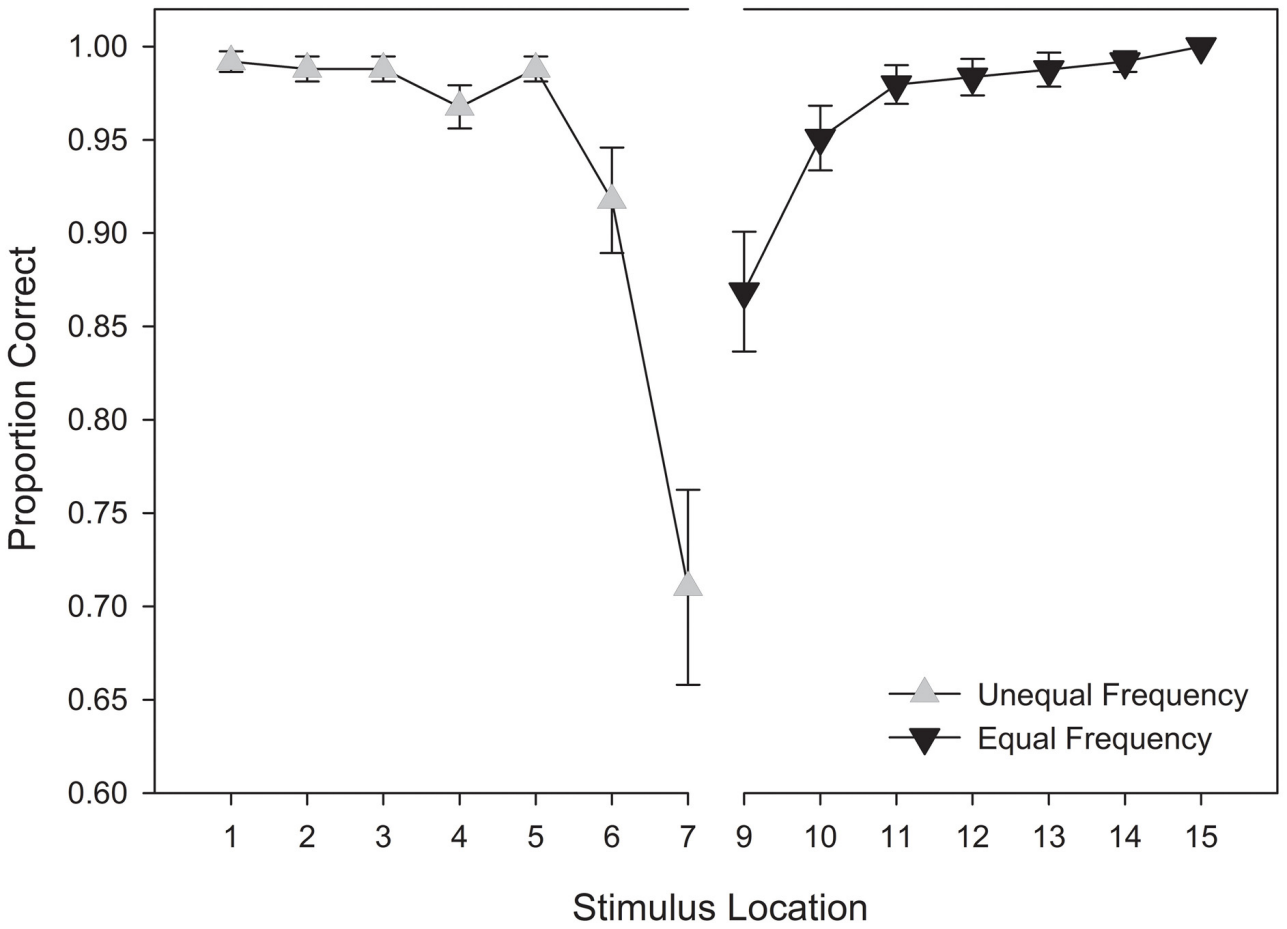
**Test categorization response accuracy in the unequal frequency, unequal distribution condition**. Stimulus values correspond to stimuli selected from the training range (i.e., stimuli 3–13). Error bars represent 1 standard error of the mean (*N* = 60).

Replicating the findings of the training phase, we observed significant main effects for stimulus distance from the category boundary, *F*_(6,174)_ = 39.887, *MSE* =0.027, *p* < 0.001, η^2^_*p*_ = 0.579, response category, *F*_(1, 29)_ = 4.516, *MSE* = 0.021, *p* = 0.042, η^2^_*p*_ = 0.135, and a significant interaction between response category and stimulus distance, *F*_(6, 174)_ = 4.082, *MSE* = 0.051, *p* = 0.036, η^2^_*p*_ = 0.134^4^. An examination of Figure 12 indicates that in each response category, the boundary-adjacent stimulus was associated with lower accuracy. However, accuracy was lower for the boundary-adjacent stimulus in the unequal frequency category in comparison to the equal frequency category.

#### Categorization response times

**Figure 13:**
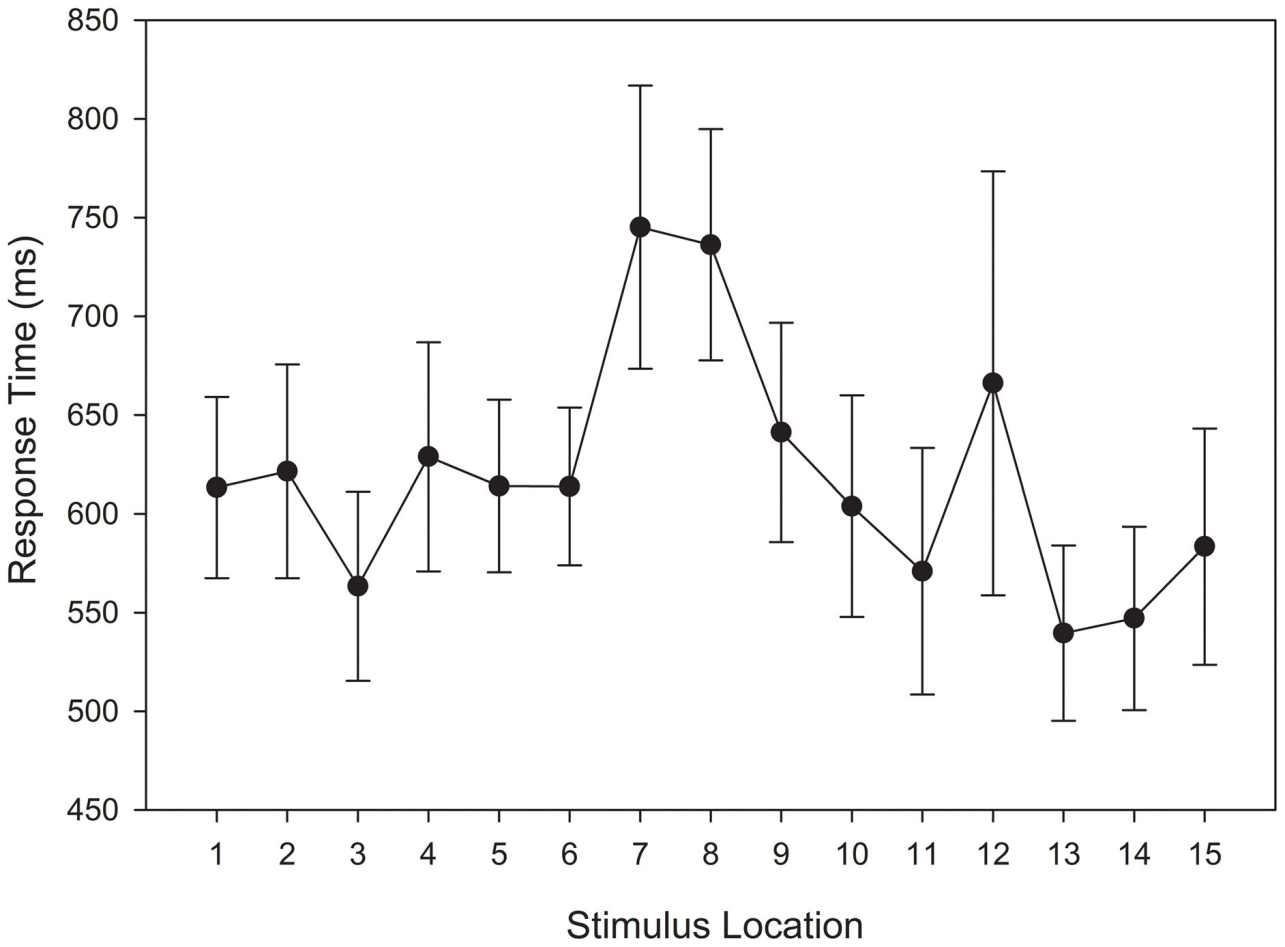
**Test categorization response time in the unequal frequency, unequal distribution condition**. Stimulus values correspond to stimuli selected from the entire stimulus range (i.e., stimuli 1–15). Error bars represent 1 standard error of the mean (*N* = 60).

A repeated measures ANOVA was conducted with categorization response time as the dependent variable. Unlike the analysis of training stimuli, no outliers met the removal criterion. Therefore, all responses were entered into the response time analysis. A main effect for stimulus distance from the category boundary reached significance, *F*_(6, 174)_ = 3.155, *MSE* = 40747.27, *p* = 0.044, η^2^_*p*_ = 0.098. Figure 13 indicates that response times for stimuli adjacent to the category boundary were relatively slower than response times for other stimuli. Again, this reflects uncertainty in category membership.

#### Category response frequencies

As in Experiment 1, we determined the frequency of “Cax” responses as a proportion of total category responses for each level along the stimulus continuum. These response frequencies were plotted across the stimulus continuum, and fitted a sigmoid function to the data.

**Figure 14:**
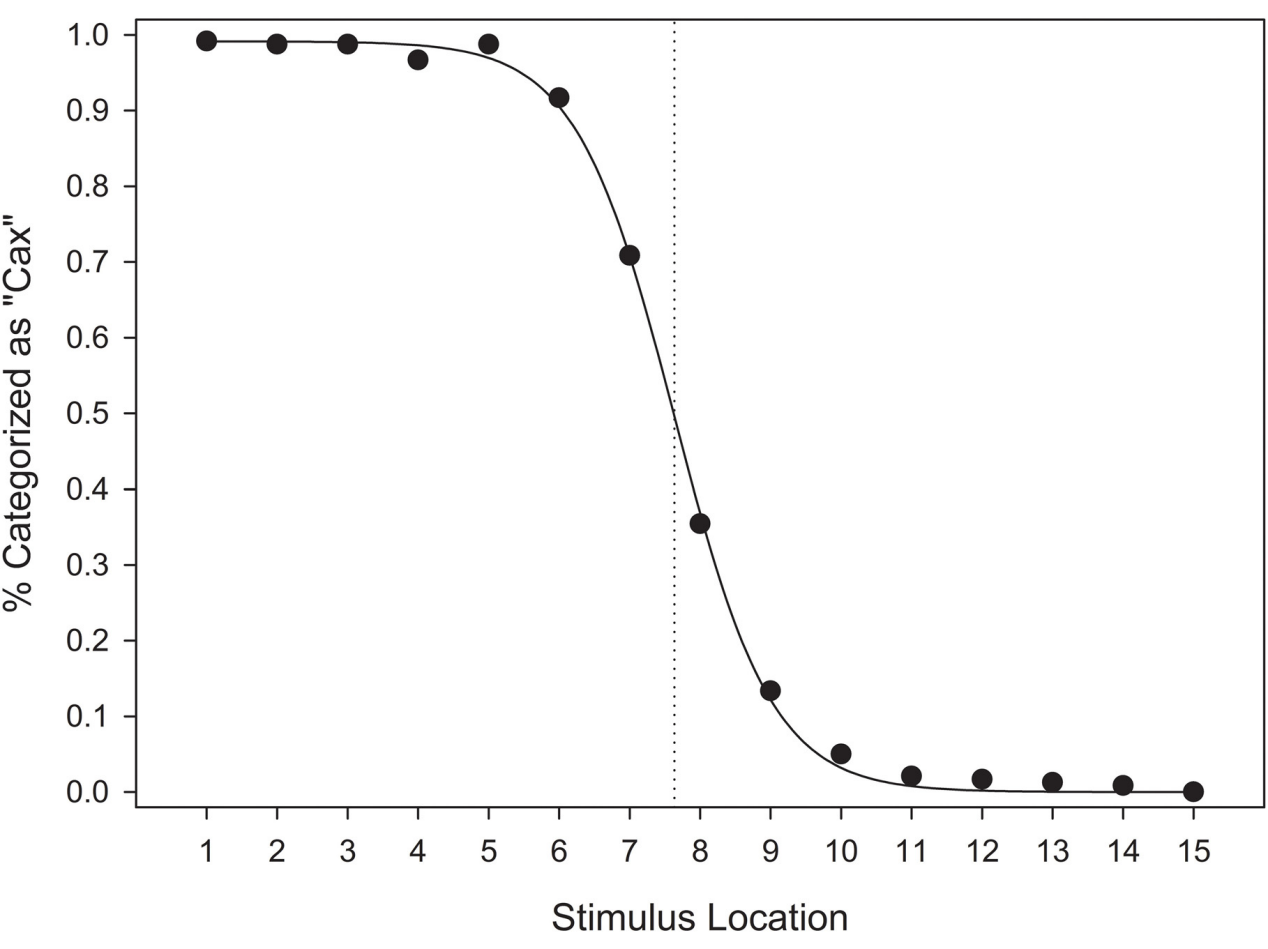
**Categorization response frequencies in the unequal frequency, unequal distribution condition**. Stimulus values correspond to stimuli selected from the entire stimulus range (i.e., stimuli 1–15). The vertical dashed line represent the estimated category boundary.

Figure 14 suggests that the category response frequencies were consistent with a sigmoidal function, and indicate that a category boundary was present near stimulus 8. A sigmoid function was found to provide an adequate fit to the data, *F*_(2, 12)_ = 8312.653, *MSE* < 0.001, *p* < 0.001, *R*^2^_*adj*_ > 0.999. Parameter estimates indicate that the point of inflection was located at 7.63. These results suggest that, in comparison to the EFED and UFED conditions in Experiment 1, the location of the category boundary was biased toward the unequal frequency category.

In order to test the possibility that the category boundary in the UFUD condition was biased due to the unequal frequency category, we conducted a follow-up analysis. We fit a sigmoid function to the response frequencies for each participant in all three of the training conditions. Then, we used two-tailed *t*-tests to compare the mean inflection points for each of the training conditions. As expected, the location of the category boundary did not differ between the EFED (*M* = 7.955, *SD* = 0.586) and UFED (*M* = 8.186, *SD* = 0.786) conditions, *t*_(58)_ = 1.283, *p* = 0.205. However, a significant difference was observed between the UFUD (*M* = 7.641, *SD* = 0.815) and UFED conditions, *t*_(58)_ = 2.636, *p* = 0.011; and a marginally significant difference was observed between the UFUD and EFED conditions, *t*_(58)_ = 1.713, *p* = 0.092.

#### Affective ratings of eeriness

**Figure 15:**
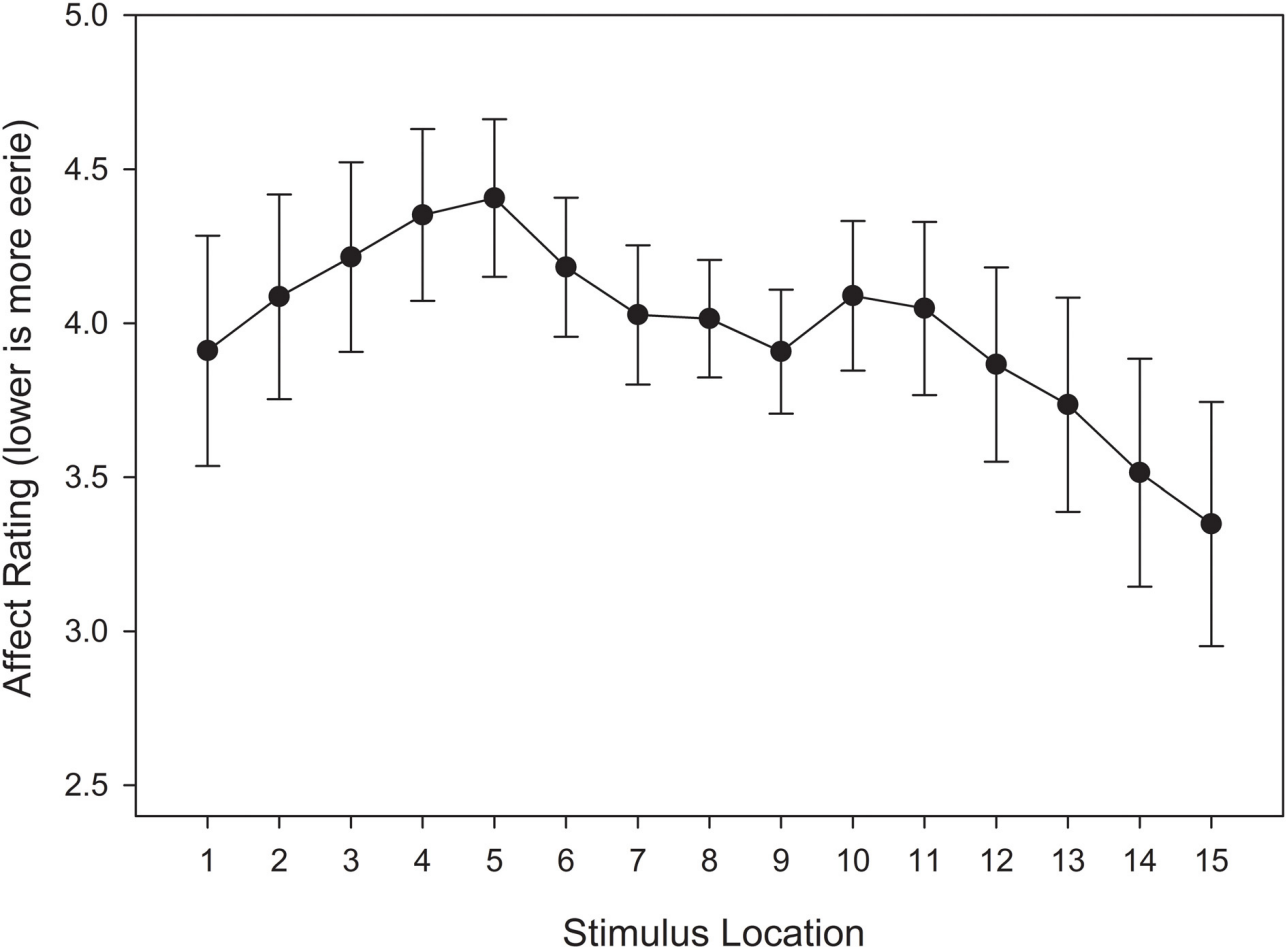
**Test affective responses (lower is more eerie) in the unequal frequency, unequal distribution condition**. Stimulus values correspond to stimuli selected from the entire stimulus range (i.e., stimuli 1–15). Error bars represent 1 standard error of the mean (*N* = 60).

A repeated measures ANOVA was conducted on affective ratings, with stimulus location (15) entered as a within-subjects variable. The main effect of stimulus location did not reach significance, *F*_(14, 406)_ = 1.387, *MSE* = 15.306, *p* = 0.258, η^2^_*p*_ = 0.046. However, a visual inspection of Figure 15 suggests a pattern that is aligned with our expectations. Specifically, there appears to be a local minimum near the category boundary, and a slope that indicates a small bias toward the unequal frequency category.

#### Correlations

Test accuracy, test response time, and affective ratings were included in a correlational analysis. Replicating the results of Experiment 1, we again observed a significant correlation between response time and categorization accuracy, *r*_(14)_ = −0.78, *p* < 0.001. Neither the correlation of eeriness ratings and accuracy, *r*_(14)_ = −0.14, *p* = 0.64, nor eeriness ratings and categorization response time reached significance, *r*_(14)_ = 0.26, *p* = 0.36. As in Experiment 1, these findings can be taken as suggesting that the response processes associated with affective ratings and categorization differ.

#### Curve fitting analysis

Next, we were interested in assessing the affective response trend. A visual inspection of Figure 15 suggests that the UFUD condition produced a distorted M-shape with a shallower region of negative affect near the category boundary, and a positive bias toward the unequal frequency category. As in Experiment 1, we fit polynomials of degree 0 through 5 (i.e., constant, linear, quadratic, cubic, quartic, and quintic) to the data, and we used the AIC as our goodness-of-fit index when comparing models.

As Table 3 suggests, constant, linear, cubic, and quintic models were rejected as they fell outside the confidence set. The model within the confidence set that was most likely to represent the data was the quartic model. This can be seen in the size of its Akaike weight, *w*_i_(*AIC*) = 0.693, meaning that it there is a 69.3% chance that it is the best model within the set. Although the quadratic model was also in the confidence set, its Akaike weight was relatively much smaller, *w*_i_(*AIC*) = 0.252. Thus, it would be reasonable to select the quartic function as best representing the data. Given the correspondence between this response pattern and the frequency-based training condition, we suggest that this result provides further evidence in support of our frequency-based exposure model of the uncanny valley phenomenon.

## Discussion

The results of Experiment 2 further qualify those obtained in Experiment 1. Although categorical performance patterns were similar to those of Experiment 1, we observed an asymmetry in categorization accuracy. Specifically, accuracy was lower for boundary-adjacent stimuli in the unequal frequency response category that was defined by fewer exemplars near the boundary, relative to the response category with more exemplars near the boundary. This suggests that the frequency training had the intended effect on categorical performance. A similar asymmetry was also observed in affective responses. However, given that measures of response times and accuracy did not correlate with affective responses, it is clear that the asymmetry in categorical perception was not the only, or even the primary, determinant of the affective asymmetry. In line with the proposed frequency-based exposure model of the UVH, we suggest that these patterns have a common cause, but that they are due to separable processes. Namely, they are both rooted in the memory traces that are encoded into long-term memory, as a result of participants' exposure to stimuli. Importantly, whereas categorical perception is the result of categorical processes which draw upon these long-term memory stores in order to determine category membership, affective responses appear to be driven by separate sub-categorical processes used to assess familiarity.

Another finding of particular importance to assessing the claims of the UVH is that the affective response pattern was in closer correspondence to the function described by Mori (1970). Specifically, we observed a slope which indicated a preference for one of the categories, and a valley region of eeriness at an intermediate region in perceptual space which was biased toward the preferred category. The primary difference between this pattern and the function described by Mori (1970) consists of the depth of the valley, and also the local affective minimum at the extreme of the preferred category. Given the similarities, we believe this pattern would provide support for a strong interpretation of UCV. These findings suggest that when a dominant reference category defined by a smaller number of high frequency exemplars is located along the same continuum as a non-dominant contrasting category defined by a larger number of low frequency exemplars, that the reference category is associated with less negative affect. Not only does such a pattern conform to the mere exposure effect (Zajonc, 1968), but it also appears to be a reasonable generalization of the UVH proposed by Mori (1970). Thus, the critical finding of Experiment 2 is that although the pattern of responses observed in categorization performance and affective responses do not co-vary, within-category differences in exemplar frequency changed participants' affective responses. Frequency effects are thereby at least as important a determinant of the UCV as categorical perception. We consider the boarder implications of these findings below.

## Concluding remarks

In the present study, we obtained patterns across multiple dependent measures that are consistent with the UVH. Our use of affective and categorization responses further allows us to draw specific conclusions concerning the relationship between these processes, thereby establishing the phenomena of the uncanny valley as well as determining its specific properties. Our analyses of cognitive responses strongly suggests that participants perceived the stimuli categorically (Cheetham et al., 2011), as evidenced in participants' categorization responses. Our analyses of affective responses revealed patterns that were consistent with the UCV function proposed by Mori (1970), providing a weak correspondence in Experiment 1, and a strong correspondence in Experiment 2. Importantly, the lack of an association between categorical and affective responses strongly suggests that affective responses cannot be understood in terms of categorical perception. Rather, categorization responses conform to patterns that would be predicted by a category boundary in terms of categorical perception, whereas affective responses conform to patterns that would be predicted on the basis of prototype or exemplar-based models. Exemplar-based models further allow for the possibility that sub-categorical properties of the stimuli influence affective responses. Thus, in addition to providing support to the uncanny valley hypothesis, our results provide an important distinction that has not, to the best of our knowledge, been adequately made within the UCV literature. We discuss this in detail below.

### Uncanny categories

Our observation that the uncanny valley is not solely the result of categorical perception stands in sharp contrast to recent accounts of the phenomenon (e.g., Burleigh et al., 2013; Yamada et al., 2013). In the categorization literature, there is still a lack of consensus about how category members are processed and stored. A pertinent distinction for the present discussion is whether an exemplar-based representation or a category boundary is used to classify stimuli. In contrast to early accounts that merely assumed that humans used definitions including necessary and sufficient conditions to classify stimuli, later accounts of categorization provided evidence that summary representations could play a critical role (Posner and Keele, 1968; Rosch and Mervis, 1975). A deficiency of these models, however, is that they fail to account for the retention of distributional properties of the stimuli (e.g., the distribution of all feline traits in domesticated cats) as well as particular instance (e.g., your pet cat). In the context of the present study, we cannot distinguish between category boundary and exemplar-based models of categorization performance. Difficulties in distinguishing between these models of categorization has been observed elsewhere when distributional properties have been manipulated experimentally (e.g., Stewart and Chater, 2002) as well as when these models are equated computationally (Ashby and Maddox, 1993). More specifically, difficulties in distinguishing these accounts on the basis of behavioral responses are likely a result of the general adaptability of participants to exemplar frequency in terms of category set size (e.g., Smith and Minda, 1998) and multiple learning systems (e.g., Nosofsky et al., 1994; Ashby et al., 1998). Our manipulation does, however, allow us to distinguish between alternative accounts of the UCV.

In the present study, we found strong evidence that supports the role of exemplar frequenies in determining affective responses. Unlike categorization responses wherein the category boundary was the primary determinant of performance, extrapolation items outside the initial training range were associated with greater negative affect relative to items within the training range. Such a finding is of considerable interest given that it goes against a number of well-established findings in the psychological literature. Specifically, end effects are observed when stimuli are presented along a stimulus continuum, and extreme items are identified more quickly and accurately then intermediate items (for a recent exemplar-based model, see Kent and Lamberts, 2005). Thus, when translated into the present study, we might imagine that negative affective responses should reach a minima in these regions. Similarly, these exemplars shared the smallest number of features with the contrasting category, meaning that there should be little feature mismatch. Our results indicate that a categorical perception model is inadequate in accounting for the results we obtained that support the UCV.

### Affect and information processing

In contrast to categorical perception accounts of the UVH, the affective responses of our participants clearly demonstrate sensitivities to the distributional properties of categories that resulted from the manipulation of exemplar frequencies (see also, Förster et al., 2010; Gillebaart et al., 2012). Distinguishing between affective and cognitive processes should be of central importance to those interested in examining the UVH. Our results are unambiguous in differentiating between categorization performance and affective response with a sharp category boundary defining the former and graded, U-shaped (parabolic) functions defining the latter within each response category. These results might be unique to the present experimental design. A limitation of the present study is that we purposefully chose not to counterbalance the order of eeriness ratings and categorization responses. Our selection of this design followed from research that affective responses are typically produced faster than more effortful cognitive processing (Bless et al., 1990; Haidt, 2001) while decision-making appears to require that alternatives have affective valence (e.g., Damasio, 1994). A straightforward account of the present findings could be that the gradation in affective responses relative to the categorization responses was a consequence of the additional processing time resulting in activation of the category structure in long-term memory. We do not consider this an important concern as we wished to demonstrate that frequency-based information influenced responses and acted as a determinant of the uncanny valley. Moreover, in a meta-analysis of the mere exposure effect conducted by Bornstein (1989), delays in preference ratings were shown to increase effect size. Thus, the results of the present study might reflect smaller effect sizes than are possible.

Interesting parallels have been drawn between the UVH and the speech perception literature (Moore, 2012). Namely, an examination of identification functions generally reveals strong evidence for categorical perception whereas identification response time can demonstrate slight gradations in response around the category boundary (Pisoni and Tash, 1974). Pisoni and Tash (1974) suggested that stimuli first undergo acoustic processing followed by phonemic processing. Depending on the speed of responses and the rate at which stimuli are presented, listeners can detect acoustic differences although there is a strong bias for identification on the basis of native linguistic distinctions. Additional evidence is provided by studies that have used ratings of stimulus typicality relative to a particular response category wherein listeners produce highly graded responses (Miller and Volaitis, 1989). For instance, Schoenherr and Logan (Schoenherr et al., 2012; Schoenherr and Logan, 2013, 2014) have examined individuals' performance when learning non-native phonemes wherein they were provided with feedback to reorganize a native continuum. These adult listeners appeared to be subjectively aware of the native category structure while producing identification responses that were influenced by the acoustic properties. Thus, if we were to have switched the order of affective and categorization responses then we might have observed more graded responses in the categorization response function and less graded responses in the affective response function. It is critical to note that this approach does not assess whether the sub-categorical information that is used to inform such categorization responses is affective in nature. We take the UVH to necessitate the inclusion of affective responses.

Our study is not the first to consider the role of categorical perception in the UCV phenomenon (Cheetham et al., 2011, 2014; Yamada et al., 2013). Cheetham et al. (2011) has proposed that Mori's hypothesis be considered “in terms of the well-established psychological empirical-theoretical framework of category perception and learning,” and further stressed the importance of “careful definition of the category boundary” (pp. 11–12). They argued that doing so would be necessary to evaluate the potential role of categorization ambiguity in eliciting negative affect. The present study is consistent with these recommendations, and to the best of our knowledge it is the first to empirically investigate the UCV phenomenon using a category-learning paradigm.

In our study, we trained participants on stimuli belonging to non-human ontological categories to which they had little or no prior exposure. We designed our training regimen to approximate the differing levels of experience that participants would have with natural categories (e.g., human or non-human animal groups). In addition to finding evidence for categorical perception, including fitting the data with logistic functions, our analysis of affective responses demonstrated frequency effects that are not consistent with categorical perception. In a similar way to exemplar-based models that have been provided in speech perception literature to account for prototype effects (Lacerda, 1995) as well as the categorization literature more generally (Medin and Schaffer, 1978; Nosofsky, 1984), we suggest that the frequency of instances is a critical determinant of the UCV. This is consistent with accounts in the categorization literature that the frequency of training stimuli will determine the representation that is acquired by participants (e.g., Smith and Minda, 1998). Rather than seeing these results as contradictory, we suggest that sharper conceptual and methodological distinctions need to be made in terms of the contributions of affective and cognitive components. If the UCV is considered to be a product of a cognitive processes, then examinations of categorization responses are not sufficient.

The present study, as well as the current literature on the UVH, leaves open a crucial question asked by researchers studying cognition and affect: what is the causal relationship between affect and cognition? Presently, we proposed two models of the uncanny valley phenomenon. Whereas the categorical perception model assumes that categorical and affective responses are integrated at some level (Stage 3), the frequency-based exposure model assumes that they are separable (Stage 2 and Stage 3 produce different responses). Both models share in the assumption that responses are a function of stimulus comparisons with representations in long-term memory. This elementary distinction leaves open still further possibilities. Elsewhere in the affective processing literature, a number of hypotheses have been put forward which merit investigation (for a review, see Cacioppo and Gardner, 1999). Perhaps more notably, models of affect in information processing have suggested that the relationship is bi-directional, such that affect also has an influence on cognition (e.g., Bless et al., 1990; Slovic et al., 2002). In general, these models assume that affect influences the spontaneous adoption of an automatic or controlled processing strategy by signaling a benign or problematic situation (Schwarz, 2002, 2010; Schwarz and Clore, 2007). In these accounts, low-cost heuristics are relied upon when encounters are expected to go smoothly, but effortful processing is recruited when obstacles are expected. In context of the UCV phenomenon, this might suggest that uncanny stimuli increase the depth of cognitive processing. This claim is supported by studies that have an association between the amount of processing and negative affect (Bless et al., 1990). If this is the case, then we would expect to find differences in memory recall for items across an uncanny perceptual continuum, such that uncanny stimuli are more distinctive in memory (Hunt and Worthen, 2006). If studies of the UVH are to make meaningful, generalizable contributions to the literature of psychology, they must clarify how the perception of categories and affect are related. The present study represents a small step in that direction.

### Conflict of interest statement

The authors declare that the research was conducted in the absence of any commercial or financial relationships that could be construed as a potential conflict of interest.

## Footnotes

^1^ A secondary analysis of arcsine transformed data revealed the same pattern as the analysis of untransformed data.

^2^ A secondary analysis of arcsine transformed data revealed the same pattern as the analysis of untransformed data.

^3^A secondary analysis of arcsine transformed data revealed the same pattern as the analysis of untransformed data.

^4^A secondary analysis of this data was conducted using arcsine transformed proportion correct values. This analysis revealed a similar pattern as the analysis of untransformed data. A change from significant to marginally significant results were observed for the main effect of response category [*F*_(1, 29)_ = 4.161, *MSE* =0.058, *p* = 0.051, η^2^_*p*_ = 0.125] and also for the interaction between response category and stimulus distance [*F*_(6, 174)_ = 2.666, *MSE* = 0.093, *p* = 0.074, η^2^_*p*_ = 0.084]. The differences between these analyses of transformed and untransformed data can likely be attributed to a reduction in observed power. Specifically, power for the main effect of response category was reduced from β = 0.538 to β = 0.505, and power for the interaction was reduced from β = 0.602 to β = 0.527.
